# Exploring the Effects of Climate Change on Child Malnutrition: A Scoping Review

**DOI:** 10.1111/jhn.70220

**Published:** 2026-02-26

**Authors:** Cecília Stanzani Klapka, Brena Barreto Barbosa, Arthur Ramalho Magalhães, Estela Barici Pereira, João Pedro Schettino, Ana Luiza Domingos, Antonio Augusto Ferreira Carioca, Bárbara Hatzlhoffer Lourenço, Leandro Martins Totaro Garcia, Michelle Cristine Medeiros Jacob, Nancy López‐Olmedo, Sávio Marcelino Gomes, Ulysses Paulino Albuquerque, Aline Martins de Carvalho

**Affiliations:** ^1^ Department of Nutrition University of São Paulo São Paulo São Paulo Brazil; ^2^ Department of Nutrition University of Fortaleza Fortaleza Brazil; ^3^ Department of Botany, Biosciences Center Federal University of Pernambuco Recife Brazil; ^4^ Federal University of São Paulo São Paulo São Paulo Brazil; ^5^ Resiclima Network International Collaboration for the Multidimensional and Interdisciplinary Study of Global Climate Change Recife Brazil; ^6^ Centre for Public Health Queen's University Belfast Belfast UK; ^7^ Department of Public Health and Nutrition Federal University of Rio Grande do Norte Natal Brazil; ^8^ Center for Population and Health Research. National Institute of Public Health Cuernavaca Mexico; ^9^ Department of Nutrition Federal University of Paraíba João Pessoa Brazil

**Keywords:** child, climate change, food security, malnutrition, obesity, undernutrition

## Abstract

**Introduction:**

Climate change is silently reshaping childhood, especially in the world's most vulnerable regions. This scoping review explores how environmental stressors—such as rising temperatures, altered rainfall patterns, droughts, and floods—affect the nutritional status of children under 5 years of age.

**Methods:**

A systematic search of four major databases yielded 1586 studies, of which 37 met the inclusion criteria.

**Results:**

Our findings reveal that climate change impacts child malnutrition primarily through indirect pathways influenced by food insecurity, disruptions in agricultural production, and deep‐rooted socioeconomic inequalities. Stunting emerged as the most frequently and severely affected outcome, while overweight and obesity were rarely addressed—highlighting important gaps in the current evidence. Socioeconomic factors such as caregiver education, rural residence, and household income were consistently identified as key variables, shaping the extent to which climate risks translate into nutritional harm. Most studies focused on countries in Sub‐Saharan Africa and South Asia, where the burden of vulnerability is greatest. Beyond documenting associations, this review draws attention to a broader reality: that child nutrition today is threatened not by a single crisis but by a web of interconnected challenges.

**Conclusion:**

As the global polycrisis unfolds, early childhood nutrition demands urgent, coordinated responses that are evidence‐based, socially just, and future‐oriented.

## Summary

- Climate change affects child malnutrition mainly through indirect socioeconomic pathways.
- Stunting is the most frequently reported and severely affected nutritional outcome.
- Food insecurity and agricultural disruption are key mediators of climate–nutrition links.
- Evidence on childhood overweight and obesity in climate studies remains scarce.
- Most evidence comes from highly vulnerable regions in Sub‐Saharan Africa and South Asia.

## 1 Introduction

Child malnutrition encompasses undernutrition (including underweight, stunting, and wasting), overweight, and micronutrient deficiencies [1]. In 2022, 22.3% of children worldwide were affected by stunting, while 5.6% were overweight. The highest rates of stunting were observed in South Asia and Sub‐Saharan Africa (around 31%). Furthermore, overweight was most prevalent in the Middle East and North Africa (10.3%), followed by Latin America and the Caribbean (8.6%), East Asia and the Pacific (8.2%), and North America (8.2%) [2]. While chronic undernutrition remains a major issue, particularly in low‐income regions, rising overweight rates reflect shifts in food systems and can be associated with increased consumption of ultra‐processed foods and reduced physical activity [3].

Climate change is among the most pressing challenges of our time, with widespread impacts on ecosystems, weather patterns, and human health [4, 5]. Beyond environmental consequences like rising temperatures and sea levels, climate change profoundly affects vulnerable populations—particularly children under 5, whose health and nutrition are highly sensitive to environmental disruptions [6]. As of 2022, nearly 1 billion children—about half of the world's total—were exposed to high or extremely high water stress, 739 million faced water scarcity, 436 million lived in areas with high water vulnerability, and 470 million were at high risk of drought [7]. These alarming numbers underscore the urgent need to address the intersection between climate change and child health.

In this context, climate change acts as a major aggravating factor by altering agricultural production patterns [8], affecting the availability and quality of food. Extreme weather events such as droughts and floods can further disrupt access to safe and nutritious food [9] while also impacting water quality and disease patterns, exacerbating existing health vulnerabilities [10]. Together, these direct and indirect effects shape a complex and precarious nutritional and health landscape for young children in a climate‐changing world.

To fully understand the extent of these impacts, it is essential to consider them within the broader context of what has recently been described as a global polycrisis [11]. The polycrisis idea encompasses the interconnected crises highlighted by the concept of syndemic crises—namely, obesity, undernutrition, and climate change—and reflects the intricate interplay of multiple global challenges, including pandemics, geopolitical tensions, and economic instability. These challenges are causally linked in ways that produce outcomes more severe than the sum of their individual effects. Through interconnected feedback mechanisms, shared stressors, and cascading effects, these crises intensify and transform one another, driving systems into unstable states. Within this framework, the nutritional and developmental threats to children arise from a tightly interconnected matrix of global systemic disruptions.

Despite growing awareness of these overlapping challenges, there is a lack of integrated evidence on how climate change directly and indirectly affects nutrition during early childhood [12]. This knowledge gap limits the capacity of public health policies to respond effectively and holistically to children's needs in a rapidly changing world.

This study aims to review the current evidence on the effects of climate change on obesity, undernutrition, and diet‐related diseases in children under 5 years of age. We analyze both direct and indirect mechanisms, with special attention to how entangled crises—within the broader polycrisis—are shaping the nutritional landscape.

## 2 Methods

This scoping review followed a previously established protocol [13]. It adhered to the guidelines outlined in the Joanna Briggs Institute Manual for Evidence Synthesis, incorporating its nine recommended steps for this type of study. Additionally, the review was conducted in accordance with the PRISMA‐ScR (Preferred Reporting Items for Systematic Reviews and Meta‐Analyses Extension for Scoping Reviews) checklist (Supporting Information: S1).

The central research question guiding this review was: *What are the effects of climate change on malnutrition in children under five years of age?* A secondary question investigated the direct and indirect mechanisms connecting these phenomena.

Inclusion and exclusion criteria were defined using the Population–Concept–Context framework recommended by the Joanna Briggs Institute. The population was defined as children under 5 years of age; the concept encompassed climate change and nutrition, and the context included studies conducted worldwide.

Four major databases were searched: Scopus, MEDLINE (PubMed), Web of Science, and Embase, which were selected for their comprehensiveness and relevance. The search was conducted in a single round between March and April 2024, with no subsequent updates. Eligible studies included original English, Portuguese, or Spanish articles with no restrictions on publication year or study design. Grey literature (e.g., theses, dissertations, reports, and books), review articles and non‐peer‐reviewed materials were excluded. While the inclusion of grey literature is often recommended in scoping reviews to broaden the scope of evidence, we chose to exclude it in order to ensure consistency in the type of data analyzed and maintain focus on studies with clearly defined and comparable methodological structures.

Search terms were organized into four categories:

- Population: Child*, Infant* or Preschool.
- Climate Change: Climate change*, Global Warming, Greenhouse gas* or Extreme Climate Event*.
- Undernutrition: Undernutrition, Underweight, Stunting, Wasting, Nutrition Deficienc*, Food Insecurit*, Food Securit* or Linear Growth.
- Obesity: Obes*, Overweight, Body Mass Index or BMI.

The asterisk (*) was used as a truncation symbol to capture variations of root words. Boolean operators were used to combine terms as follows: *(Population AND Climate Change AND Obesity)*; *(Population AND Climate Change AND Undernutrition)*; and *(Population AND Climate Change AND Obesity AND Undernutrition)* (Supporting Information S2: Table 1).

The screening process comprised three phases: title screening, abstract screening, and full‐text review. The reviewers performed all steps independently to minimize bias, with discrepancies resolved by a third reviewer. During the title and abstract screening, studies were included only if they addressed all three core components: (1) population of children under five, (2) climate change, and (3) malnutrition (obesity and/or undernutrition).

In the full‐text screening stage, we verified whether the study population matched the target age group, whether full‐text access was available, and whether the study met all inclusion criteria. Studies that did not meet these conditions were excluded.

Data extraction was conducted using a standardized form by two independent reviewers. The extracted information included the study title, first author, year of publication, authors' institutional affiliation, study location (country/region), study design, study population, journal, study objectives, study period, presence of conflicts of interest in the study, primary variables used in the studies for climate change and child malnutrition and the key strengths of these variables as perceived by the reviewers. This interpretation was based on the studies themselves and on the definitions of the primary variables.

The definitions used for wasting, stunting, undernutrition, underweight, and overweight were based on the World Health Organization (WHO) classification. Wasting is defined as a weight‐for‐height *z*‐score below −2 standard deviations (SDs) from the median of the WHO Child Growth Standards; stunting is defined as height‐for‐age *z*‐score below −2 SD; underweight is defined as weight‐for‐age *z*‐score below −2 SD; undernutrition refers to a condition encompassing wasting, stunting, and underweight; and overweight is defined as weight‐for‐height *z*‐score above +2 SD from the median; and obesity is defined as weight‐for‐height *z*‐score above +3 SD from the median [14].

Additionally, the review synthesized data on the relationships between climate change and nutritional outcomes. A structured table was created to map direct and indirect associations between climate‐related variables and indicators of undernutrition and obesity (see Supporting Information S3: Table 2). This mapping helped identify patterns, mechanisms, and potential gaps in the literature.

Variables were further categorized into three domains: Environmental, Socioeconomic/Demographic, and Health. For instance, ‘Increase in intensity and variability of precipitation patterns’ encompassed excessive rainfall, cumulative lifetime rainfall exposure, prenatal rainfall exposure, precipitation extremes, and delayed monsoons. Some variables were grouped and renamed by the authors, with consensus among all team members. This classification facilitated standardization and improved the clarity of variable interpretation.

We developed a bubble map based on the organized data to visually represent the relationships between climate change and child nutrition outcomes. It illustrates the number of studies supporting each association between the variables – the greater the number of studies, the larger the bubble size and the stronger the colour intensity. This number reflects only the quantity of studies, as the scoping review did not assess the quality of the articles, meaning all studies were assigned equal weight. The map highlights how various proxy indicators of climate change are associated with nutritional conditions, particularly undernutrition and obesity.

All authors rigorously reviewed the bubble map to ensure its accuracy, consistency, and analytical value. This visual representation was considered a robust instrument for synthesizing and interpreting the complex interactions between climate change and child nutrition following revisions and consensus.

## 3 Results

**Figure 1:**
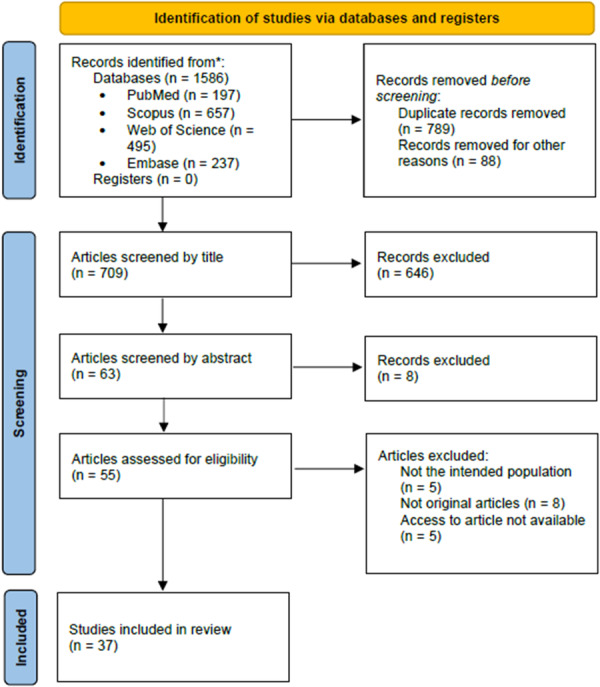
Summary of selection process using PRISMA flowchart. *Source:* The authors (2026).

The search returned 1586 articles. After removing 789 duplicates and excluding 88 non‐original articles, 709 documents remained for screening. During the screening process, 646 documents were excluded based on title review, 8 during abstract screening, and 18 in the full‐text review phase due to inaccessibility of full‐text or discrepancies with the inclusion criteria (Figure 1). The final selection consisted of 37 articles [15, 16, 17, 18, 19, 20, 21, 22, 23, 24, 25, 26, 27, 28, 29, 30, 31, 32, 33, 34, 35, 36, 37, 38, 39, 40, 41, 42, 43, 44, 45, 46, 47, 48, 49, 50, 51].

The studies exclusively employed quantitative analyses and were published between 2011 and 2023. The lead authors of most studies (*n* = 30) were affiliated with high‐income countries such as the United States and the United Kingdom, despite the majority of the research being conducted in low‐income and lower‐middle‐income countries (*n* = 33). Although many of these studies were conducted on a multinational scale, the primary focus was on countries in Sub‐Saharan Africa and South Asia, covering both urban and rural settings. The age groups of children studied varied, including segments such as 6–59 months and 1–5 years. Infants under 6 months or 1 year were excluded in some studies because their nutrition is predominantly influenced by breastfeeding and complementary feeding, making them less directly affected by environmental changes and dietary intake (Table 1). All studies addressed undernutrition [15, 16, 17, 18, 19, 20, 21, 22, 23, 24, 25, 26, 27, 28, 29, 30, 31, 32, 33, 34, 35, 36, 37, 38, 39, 40, 41, 42, 43, 44, 45, 46, 47, 48, 49, 50, 51], while only one article also examined overweight [23] and another one also mentioned anaemia [32].

**Table 1:** Summary of the main characteristics of the 37 articles included in this scoping review.

	% (*n*)
Study design	
Cross‐sectional	45.9 (17)
Longitudinal	43.2 (16)
Observational	5.4 (2)
Ecological	2.7 (1)
Descriptive	2.7 (1)
Study population	
Children in utero	2.7 (1)
Children aged 3–36 months	2.7 (1)
Children aged 6–59 months	10.8 (4)
Children aged 7–60 months	2.7 (1)
Children under 3 years	2.7 (1)
Children aged 1–5 years	5.4 (2)
Children under 5 years	72.9 (27)
Nutritional outcomes	
Stunting	94.5 (35)
Wasting	48.6 (18)
Underweight	35.1 (13)
Overweight	2.7 (1)
Anaemia	2.7 (1)
Main environmental variables	
Drought	18.9 (7)
Flood	8.1 (3)
Increase in intensity and variability of precipitation	43.2 (16)
Increase in frequency of temperature‐related extreme events	27 (10)
PM2.5 exposure	2.7 (1)
Climate change	5.4 (2)
Income status of the country of the first author	
Low income	5.4 (2)
Low‐middle income	5.4 (2)
Upper‐middle income	8.1 (3)
High income	81.1 (30)
Income status of the countries analyzed in the study	
Low income	64.9 (24)
Low‐middle income	24.3 (9)
Upper‐middle income	2.7 (1)
High income	2.7 (1)
Mixed	5.4 (2)
Geographical area of the countries analyzed in the study	
Africa	56.8 (21)
Asia	29.7 (11)
Europe	0 (0)
North America	0 (0)
South America	2.7 (1)
Oceania	0 (0)
Mixed	10.8 (4)
Space setting of the population analyzed in the study	
Urban	5.4 (2)
Rural	43.2 (16)
Both	51.4 (19)
Results disaggregated by the age of the population analyzed in the study	
Yes	37.8 (14)
No	62.2 (23)

An analysis of the methods reveals a predominance of cross‐sectional design [17, 18, 19, 23, 25, 29, 32, 35, 36, 38, 42, 43, 45, 46, 47, 50, 51] and longitudinal studies based on secondary data [15, 16, 20, 21, 22, 24, 26, 30, 34, 39, 40, 42, 44, 49]. Only four studies employed prospective modelling [28, 30, 31, 39] and spatial analysis [27, 36, 41, 47]. The diversity in environmental variables used and the heterogeneity of socioeconomic and health indicators hinder direct comparisons. The methodological plurality underscores the lack of standardization in indicators and analytical strategies, reducing the comparability of findings.

The studies examined a diverse set of variables to explore the links between climate change and child nutrition:

- Anthropometric: Height‐for‐age, weight‐for‐height, and weight‐for‐age, used to assess stunting, wasting and underweight, respectively.
- Environmental: Temperature, precipitation, Normalized Difference Vegetation Index (NDVI), exposure to droughts and floods, Standardized Precipitation Evapotranspiration Index and future climate projections. These variables were analyzed using methods such as the assessment of monthly averages for precipitation, mean and maximum temperatures, periods of drought and climate scenarios for the future (as detailed in Supporting Information S3: Table 2).
- Socioeconomic: Household size, wealth/income, urban or rural residence status, mother's education, access to water and sanitation, and the primary occupation of household members.
- Health‐related: Immunization status, birth weight, breastfeeding duration, presence of diseases (e.g., fever, diarrhoea), and anaemia status.
- Nutritional: Food security, nutrient intake (such as protein, zinc, and vitamin A), caloric intake, and dietary diversity.

The review revealed that climate change does indeed affect malnutrition in children under 5 years of age, particularly in relation to stunting and wasting, which were the most frequently reported outcomes (with 35 articles addressing stunting and 18 addressing wasting). While some studies suggest a relationship between climate change and overweight, this association was identified in only one article.

Among the growth indicators, height‐for‐age was identified as the most affected by these climatic changes [15, 16, 18, 19, 21, 22, 23, 24, 26, 27, 28, 29, 30, 31, 34, 35, 36, 40, 42, 43, 46, 47, 49, 50, 51]. Regions experiencing increased temperatures [16, 22, 23, 24, 26, 42, 43, 46, 47] and altered precipitation patterns [5, 16, 18, 19, 21, 22, 23, 24, 26, 27, 34, 40, 43, 47] demonstrated a notably higher prevalence of growth delays, commonly referred to as stunting, which serves as a key indicator of chronic malnutrition and its long‐term effects on child health and development.

**Figure 2:**
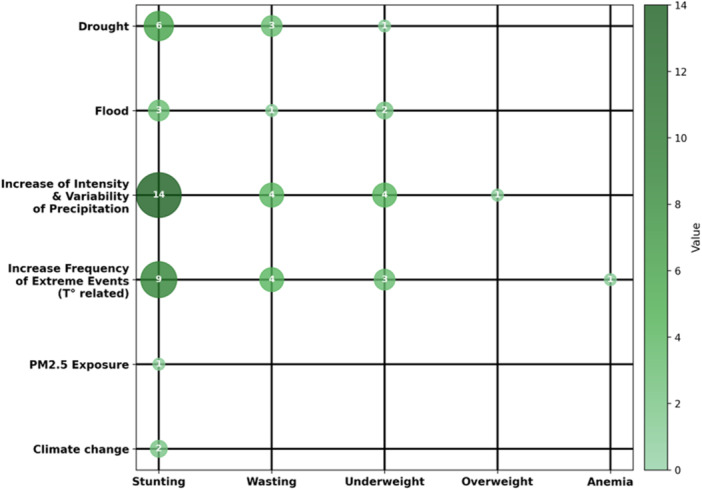
Bubble map linking environmental variables and nutrition outcomes. *Source:* The authors (2026).

The bubble map (Figure 2) shows clearly that all observed relationships between environmental variables and nutrition outcomes move in the same direction, meaning both increase or decrease together. Specifically, stunting exhibits this type of relationship with all environmental variables. Therefore, according to the findings of this review, greater climate change is associated with an increased likelihood of a child developing stunting.

For wasting and underweight, this pattern is observed only in relation to drought [15, 16, 40], flood [29, 35], increase in intensity and variability of precipitation patterns [21, 26, 34, 40, 41, 42, 47] and increased frequency of temperature‐related events [22, 25, 26, 41, 46]. In other words, these specific climate change proxies are associated with a greater likelihood of these two forms of malnutrition.

Overweight was positively correlated with increased intensity and variability of precipitation patterns, indicating a potential link between climate change and weight gain in children under five. However, this relationship is found in only one study in our review [23]. Similarly, anaemia was positively associated with the increased frequency of temperature‐related extreme events, but again, this connection is mentioned in only one study [32].

No inverse relationship between climate change and nutrition outcomes was found. There were no studies that did not find a lack of association. In other words, this review confirms a correlation in which greater climate change leads to more negative nutritional outcomes and demonstrates that there is no scenario in which these changes would reduce malnutrition.

Also, this review revealed that the effects of climate change on the nutritional status of children under 5 years of age occur through indirect pathways. In other words, climatic variables do not act in isolation but are influenced by socioeconomic and health‐related factors that intensify their impact and increase the likelihood of adverse nutritional outcomes. For example, families living in poverty are more vulnerable to the consequences of extreme weather events, such as heavy rainfall or droughts, as they often lack the resources needed to adapt. Thus, climate change indirectly contributes to an increase in malnutrition. In other words, as climate change intensifies, malnutrition tends to worsen. This relationship and its outcomes are influenced by both socioeconomic and health‐related factors.

The most frequently investigated socioeconomic variables across the studies were rural residence (*n* = 8), caregiver education (*n* = 9), socioeconomic vulnerability (*n* = 7), and household income (*n* = 8). Among these, rural location emerged as a consistent variable that impacts the relationship between climate change and child undernutrition. Specifically, rural residence was associated with a higher prevalence of stunting in contexts affected by drought [49, 50], increased intensity and variability of precipitation patterns [22, 43], and the rising frequency of temperature‐related extreme events [22, 43]. Similar associations were observed for wasting [22, 25] and underweight [25] under conditions of elevated heat extremes.

Regarding caregiver education, it affects the relationship between stunting and drought [15, 36, 49], flood [36], increased intensity and variability of precipitation patterns [18, 19, 23], and increased frequency of temperature‐related extreme events [23]. For wasting, caregiver education is related to the relationship with drought [15], increased intensity and variability of precipitation patterns [26], and increased frequency of temperature‐related extreme events [26].

In terms of vulnerability, it influences the relationship with stunting in connection with drought [16, 50], increased intensity and variability of precipitation patterns [16, 18, 19, 26], and increased frequency of temperature‐related extreme events [16, 26]. For wasting, vulnerability impacted the relationship with increased intensity and variability of precipitation patterns [26] and increased frequency of temperature‐related extreme events [26]. In all these cases, the more education the parents in the household have, the greater the chance of protecting or mitigating the effects of climate change, leading to better nutritional outcomes for the children. Vulnerability was not always presented as a standalone variable, but rather as a composite condition derived from multiple socioeconomic and environmental indicators. This variable was commonly operationalized through proxies such as rural residence, access to infrastructure (e.g., clean water, sanitation, electricity), and livelihood dependence on climate‐sensitive sectors such as agriculture.

As for income, it is related to stunting in connection with drought [36], flood [29, 36], increased intensity and variability of precipitation patterns [23, 26], and increased frequency of temperature‐related extreme events [23, 26, 45]. For wasting, income affects the relationship with increased intensity and variability of precipitation patterns [26] and increased frequency of temperature‐related extreme events [26]. Lastly, for underweight, income influences the relationship observed with increased intensity and variability of precipitation patterns [41] and increased frequency of temperature‐related extreme events [41]. In all these cases, the higher the income, the greater the chance of better protection against climate change, leading to a lower likelihood of food insecurity and negative nutritional outcomes.

## 4 Discussion

Our findings, based on a comprehensive scoping review of 37 original research articles conducted in diverse global contexts, revealed a lack of clearly described direct effects of climate change on child malnutrition. Instead, we identified indirect pathways—particularly influenced by socio‐economic factors—through which climate events such as droughts, floods, and excessive rainfall affect nutritional outcomes. Across studies, socioeconomic vulnerability, including poverty, limited caregiver education, and inadequate living conditions, emerged as the central pathway through which climate‐related shocks influence child nutrition. These findings highlight the many aspects involved in this relationship.

Several mediators are typically involved when examining the link between climate change and malnutrition. One of the primary mechanisms identified is food security and agricultural productivity. In Sub‐Saharan Africa, increased temperatures and altered precipitation patterns have been associated with reduced crop yields, directly impacting food availability and contributing to malnutrition [52]. Similarly, in India, excessive monsoon rainfall can disrupt agricultural cycles, leading to food shortages and an increased risk of undernutrition among children [53].

These dynamics are most evident in settings highly dependent on climate‐sensitive agricultural systems, namely Sub‐Saharan Africa and South Asia. In these areas, extreme weather events—such as droughts and floods—combined with rising temperatures exacerbate food insecurity and child malnutrition [47, 54]. Communities in these regions, which are highly dependent on agriculture, face difficulties adapting to changing climatic conditions. This leads to reduced food availability, lower dietary diversity, and increased rates of stunting, wasting, and anaemia among children [25, 48].

Another important aspect is the socio‐economic relationship between income levels and child malnutrition. Studies have demonstrated a direct link between lower‐income households and being more vulnerable to climate disruptions. Climate change exacerbates these disparities by reducing agricultural yields and decreasing household income, particularly in rural and farming‐dependent populations [55]. As food production declines, supply shortages drive up prices, limiting access to food and increasing malnutrition risk among children in low‐income families [56]. Thus, climate change is a significant mediator, deepening pre‐existing socio‐economic inequalities and worsening food insecurity and nutritional outcomes [57].

Other socio‐economic factors, such as caregiver education and social vulnerability—including access to sanitation, electricity, and proximity to water—are closely linked to low‐income households and further mediate the effects of climate change on child nutrition. For instance, maternal illiteracy and limited access to clean water and high‐polluting cooking fuels—such as kerosene, coal, charcoal, wood, straw, crop waste, and dung [28] —are more prevalent in low‐income families and are associated with higher rates of stunting, wasting, and underweight in children in rural areas of South and Southeast Asia [58]. Maternal education, in particular, is a key determinant of child nutrition, as it influences dietary practices, health‐seeking behaviours, and the ability to adapt to climate variability [59].

Despite the well‐established relationship between climate change and child undernutrition, its association with being overweight remains unclear. Only one study included in this review found a correlation between increased precipitation variability and childhood obesity [23]. However, emerging evidence suggests that climate change affects food systems by reducing dietary diversity and nutrient quality, which are crucial for preventing the double burden of malnutrition—where undernutrition coexists with overweight and obesity. The industrialization and globalization of food systems have contributed to the widespread availability of high‐energy, low‐nutrient foods, which are linked to rising obesity rates, even as undernutrition persists due to inadequate access to nutritious foods, including for children [60].

Among the nutritional consequences of climate change, this review identified stunting as the most affected indicator. This can be explained by linear growth reflecting cumulative nutritional status over time, making it a sensitive marker of adverse environmental conditions that affect long‐term child nutrition [61]. Moreover, once a population demonstrates a significant prevalence of stunting, this is a highly stable indicator, one that does not fluctuate easily due to short‐term interventions, unlike underweight. The sensitivity of stunting to climate change appears to result from its dependence on a broad range of socio‐environmental determinants, including food security, economic conditions, and health status—all of which are influenced by climate variability [62, 63].

Adaptive public policies are essential to mitigate the impacts of climate change on child malnutrition, particularly those aimed at enhancing agricultural productivity to strengthen food security. In northwestern Cambodia, for instance, strategies supporting smallholder farmers include climate‐resilient agricultural practices, investments in water management, and access to reliable climate information. These measures help farmers adapt to changing conditions, improve crop yields, and ensure food availability [64]. Tailoring interventions to specific regional needs is also crucial. In South Asia, improving sanitation infrastructure and expanding educational opportunities for women can reduce vulnerability to climate‐driven undernutrition [18]. Moreover, understanding how local temperature and rainfall patterns affect agriculture and nutrition can inform more effective, targeted responses [63]. Policymakers can reinforce interventions that consider maternal education and promote climate‐adaptive strategies—such as drought and flood resilience—to mitigate child malnutrition and build more resilient food systems.

This study has several limitations. First, despite being a scoping review, grey literature and unpublished studies were not included. We focused solely on original research articles because we aimed to understand the relationship between climate change and child nutrition. Second, the methodological quality of the included studies was not assessed, which may result in the inclusion of studies with less robust methods; however, this is an inherent characteristic of scoping reviews. Third, some variables were grouped into broader categories to streamline analysis and presentation, which may have introduced bias. Nonetheless, the authors reviewed and validated this process to minimize distortions.

A noteworthy limitation of the available evidence is its geographical concentration, since most studies were conducted in Sub‐Saharan Africa and South Asia. This reflects both the heightened vulnerability of these regions to climate shocks and a research bias toward low‐income and lower‐middle‐income contexts. Furthermore, 43% of the studies analyzed rural populations exclusively, which may limit the applicability of findings to urban or peri‐urban settings. Although rural areas are more exposed to climate‐related risks due to their dependence on agriculture, further research is needed in urban and high‐income settings, where different pathways (such as air pollution, food system transitions and socioeconomic disparities) may influence child nutrition.

Despite these limitations, this scoping review makes an original contribution to the literature by offering a nutrition‐centred synthesis of the effects of climate change on early childhood. Rather than treating nutrition as a secondary outcome, this study places malnutrition in children under 5 years of age at the core of the analysis and systematically maps specific climate‐related exposures to distinct nutritional outcomes. The findings highlight how climate‐related stressors operate in conjunction with socioeconomic and health conditions, shaping persistent patterns of nutritional vulnerability, particularly stunting. In addition, the structured visual mapping approach employed enables the identification of consistent patterns and critical gaps in the evidence, most notably the limited attention to overweight and obesity, thereby advancing understanding beyond descriptive associations and informing more targeted, climate‐sensitive nutrition research and policy.

## 5 Conclusion

This scoping review offers novel insights into the complex interplay between climate change and child malnutrition. By consolidating fragmented evidence, we demonstrate that climatic stressors—rising temperatures, altered rainfall patterns, droughts, and floods—are consistently associated with adverse nutritional outcomes in children under 5 years of age, with stunting emerging as the most frequently and severely affected indicator.

Our findings become even more salient when considered through the lens of the global polycrisis—a framework that emphasizes the interconnected and compounding nature of contemporary systemic shocks. In this context, child malnutrition does not occur in isolation but is embedded within a cascade of interlinked disruptions across food systems, public health, economic stability, and environmental degradation. The role of socioeconomic mediators—such as poverty, caregiver education, and rural residence—illuminates how climate shocks intersect with long‐standing structural vulnerabilities, reinforcing health inequities and amplifying risk in early childhood.

These insights underscore the need to translate evidence into practice, guiding policymakers and practitioners toward interventions that are context‐specific, equity‐oriented, and systemically integrated. We call for urgent integrated policy responses that are not only climate‐sensitive and nutrition‐informed but also explicitly designed to address the converging pressures of a destabilizing global system.

## Author Contributions

**Cecília Stanzani Klapka:** conceptualization, data curation, investigation, methodology, writing – original draft. **Brena Barreto Barbosa:** data curation, investigation, methodology, writing – original draft. **Arthur Ramalho Magalhães:** data curation, investigation, methodology. **Antonio Augusto Ferreira Carioca:** conceptualization. **Bárbara Hatzlhoffer Lourenço:** conceptualization. **Leandro Martins Totaro Garcia:** conceptualization. **Nancy López‐Olmedo:** conceptualization. **Sávio Marcelino Gomes:** conceptualization. **Ulysses Paulino Albuquerque:** conceptualization. **Aline Martins de Carvalho:** conceptualization, supervision, guarantor. All authors: validation, writing – review and editing.

## Ethics Statement

This study is a review of published data and did not require ethics approval.

## Conflicts of Interest

The authors declare no conflicts of interest.

## Supporting information

PRISMA ScR Checklist Klapka 2025.

Supplementary 1 Search Strategy Klapka 2025.

Supplementary Table 2.

## Data Availability

The data that support the findings of this study are available from the corresponding author upon reasonable request.

## References

[1] World Health Organization , Malnutrition: Fact Sheets [Internet] (World Health Organization, 2024), https://www.who.int/news-room/fact-sheets/detail/malnutrition.

[2] United Nations Children's Fund (UNICEF), World Health Organization, World Bank, *Joint Child Malnutrition Estimates* [Internet] (UNICEF, 2023), https://datatopics.worldbank.org/child-malnutrition/.

[3] B. M. Popkin , C. Corvalan , and L. M. Grummer‐Strawn , “Dynamics of the Double Burden of Malnutrition and the Changing Nutrition Reality,” Lancet 395, no. 10217 (2020): 65–74. 31852602 10.1016/S0140-6736(19)32497-3 PMC7179702

[4] Y. Malhi , J. Franklin , N. Seddon , et al., “Climate Change and Ecosystems: Threats, Opportunities and Solutions,” Philosophical Transactions of the Royal Society, B: Biological Sciences 375, no. 1794 (2020): 20190104. 10.1098/rstb.2019.0104 PMC7017779 31983329

[5] Q. Zhao , P. Yu , R. Mahendran , et al., “Global Climate Change and Human Health: Pathways and Possible Solutions,” Eco‐Environment & Health 1, no. 2 (2022): 53–62. 38075529 10.1016/j.eehl.2022.04.004 PMC10702927

[6] D. Helldén , C. Andersson , M. Nilsson , K. L. Ebi , P. Friberg , and T. Alfvén , “Climate Change and Child Health: A Scoping Review and an Expanded Conceptual Framework,” Lancet Planetary Health 5, no. 3 (2021): e164–e175. 33713617 10.1016/S2542-5196(20)30274-6

[7] United Nations Children's Fund (UNICEF), *The Climate‐Changed Child: A Children's Climate Risk Index Supplement* [Internet] (UNICEF, 2023), https://www.unicef.org/reports/climate-changed-child#download-the-report.

[8] W. Leal Filho , A. F. F. Setti , U. M. Azeiteiro , et al., “An Overview of the Interactions Between Food Production and Climate Change,” Science of the Total Environment 838, no. Pt 3 (2022): 156438. 35660578 10.1016/j.scitotenv.2022.156438

[9] R. A. Duchenne‐Moutien and H. Neetoo , “Climate Change and Emerging Food Safety Issues: A Review,” Journal of Food Protection 84, no. 11 (2021): 1884–1897. 34185849 10.4315/JFP-21-141

[10] P. Van de Vuurst and L. E. Escobar , “Climate Change and Infectious Disease: A Review of Evidence and Research Trends,” Infectious Diseases of Poverty 12, no. 1 (2023): 51. 37194092 10.1186/s40249-023-01102-2 PMC10186327

[11] M. Lawrence , T. Homer‐Dixon , S. Janzwood , J. Rockström , O. Renn , and J. F. Donges , “Global Polycrisis: The Causal Mechanisms of Crisis Entanglement,” Global Sustainability 7, no. e6 (2024): 1–16.

[12] A. M. de Carvalho , L. M. T. Garcia , B. H. Lourenço , et al., “Exploring the Nexus Between Food Systems and the Global Syndemic Among Children Under Five Years of Age Through the Complex Systems Approach,” International Journal of Environmental Research and Public Health 21, no. 7 (2024): 893. 39063469 10.3390/ijerph21070893 PMC11276875

[13] C. S. Klapka , B. B. Barbosa , A. R. Magalhães , et al., “Exploring the Effects of Climate Change on Child Malnutrition: Protocol for a Scoping Review,” BMJ Open 14, no. 12 (2024): e090285. 10.1136/bmjopen-2024-090285 PMC11683906 39725443

[14] WHO Multicentre Growth Reference Study Group , WHO Child Growth Standards: Length/Height‐for‐Age, Weight‐for‐Age, Weight‐for‐Length, Weight‐for‐Height and Body Mass Index‐for‐Age: Methods and Development [Internet] (World Health Organization, 2006), https://www.who.int/publications/i/item/924154693X.

[15] A. Dimitrova , “Seasonal Droughts and the Risk of Childhood Undernutrition in Ethiopia,” World Development 141 (2021): 105417.

[16] H. Freudenreich , A. Aladysheva , and T. Brück , “Weather Shocks Across Seasons and Child Health: Evidence From a Panel Study in the Kyrgyz Republic,” World Development 155 (2022): 105801.

[17] S. Blom , A. Ortiz‐Bobea , and J. Hoddinott , “Heat Exposure and Child Nutrition: Evidence From West Africa,” Journal of Environmental Economics and Management 115 (2022): 102698.

[18] K. McMahon and C. Gray , “Climate Change, Social Vulnerability and Child Nutrition in South Asia,” Global Environmental Change 71 (2021): 102414. 34898861 10.1016/j.gloenvcha.2021.102414 PMC8653856

[19] K. Grace , F. Davenport , C. Funk , and A. M. Lerner , “Child Malnutrition and Climate in Sub‐Saharan Africa: An Analysis of Recent Trends in Kenya,” Applied Geography 35, no. 1–2 (2012): 405–413.

[20] K. Johnson and M. E. Brown , “Environmental Risk Factors and Child Nutritional Status and Survival in a Context of Climate Variability and Change,” Applied Geography 54 (2014): 209–221.

[21] B. C. Thiede and C. Gray , “Climate Exposures and Child Undernutrition: Evidence From Indonesia,” Social Science & Medicine 265 (2020): 113298. 32932006 10.1016/j.socscimed.2020.113298 PMC7738425

[22] E. van der Merwe , M. Clance , and E. Yitbarek , “Climate Change and Child Malnutrition: A Nigerian Perspective,” Food Policy 113 (2022): 102281.

[23] A. Ngwira , “Climate and Location as Determinants of Childhood Stunting, Wasting, and Overweight: An Application of Semiparametric Multivariate Probit Model,” Nutrition 70 (2020): 100010. 10.1016/j.nutx.2020.100010 34301371

[24] S. Hagos , T. Lunde , D. H. Mariam , T. Woldehanna , and B. Lindtjørn , “Climate Change, Crop Production and Child Undernutrition in Ethiopia; a Longitudinal Panel Study,” BMC Public Health 14, no. 1 (2014): 884. 25163522 10.1186/1471-2458-14-884 PMC4158109

[25] R. E. Baker and J. Anttila‐Hughes , “Characterizing the Contribution of High Temperatures to Child Undernourishment in Sub‐Saharan Africa,” Scientific Reports 10, no. 1 (2020): 18796. 33139856 10.1038/s41598-020-74942-9 PMC7606522

[26] S. Dasgupta and E. J. Z. Robinson , “Climate, Weather and Child Health in Burkina Faso,” Australian Journal of Agricultural and Resource Economics 67 (2023): 576–602.

[27] D. Lopez‐Carr , K. M. Mwenda , N. G. Pricope , et al., “Climate‐Related Child Undernutrition in the Lake Victoria Basin: An Integrated Spatial Analysis of Health Surveys, NDVI, and Precipitation Data,” IEEE Journal of Selected Topics in Applied Earth Observations and Remote Sensing 9, no. 6 (2016): 2830–2835.

[28] A. Dimitrova , G. Marois , G. Kiesewetter , et al., “Projecting the Impact of Air Pollution on Child Stunting in India—Synergies and Trade‐Offs Between Climate Change Mitigation, Ambient Air Quality Control, and Clean Cooking Access,” Environmental Research Letters 17 (2022): 104004.

[29] J. M. Rodriguez‐Llanes , S. Ranjan‐Dash , O. Degomme , A. Mukhopadhyay , and D. Guha‐Sapir , “Child Malnutrition and Recurrent Flooding in Rural Eastern India: A Community‐Based Survey,” BMJ Open 1, no. 2 (2011): e000109. 10.1136/bmjopen-2011-000109 PMC3208901 22080535

[30] S. J. Lloyd , R. S. Kovats , and Z. Chalabi , “Climate Change, Crop Yields, and Undernutrition: Development of a Model to Quantify the Impact of Climate Scenarios on Child Undernutrition,” Environmental Health Perspectives 119 (2011): 1817–1823. 21844000 10.1289/ehp.1003311 PMC3261974

[31] S. J. Lloyd , M. Bangalore , Z. Chalabi , et al., “A Global‐Level Model of the Potential Impacts of Climate Change on Child Stunting via Income and Food Price in 2030,” Environmental Health Perspectives 126 (2018): 097007. 30256154 10.1289/EHP2916 PMC6375465

[32] Y. Zhu , C. He , A. Gasparrini , et al., “Global Warming May Significantly Increase Childhood Anemia Burden in Sub‐Saharan Africa,” One Earth 6, no. 10 (2023): 1388–1399. 37904727 10.1016/j.oneear.2023.09.003 PMC7615260

[33] B. Straight , X. Qiao , D. Ngo , et al., “Epigenetic Mechanisms Underlying the Association Between Maternal Climate Stress and Child Growth: Characterizing Severe Drought and Its Impact on a Kenyan Community Engaging in a Climate Change‐Sensitive Livelihood,” Epigenetics 17, no. 13 (2022): 2421–2433. 36242778 10.1080/15592294.2022.2135213 PMC9665148

[34] I. Mank , K. Belesova , J. Bliefernicht , et al., “The Impact of Rainfall Variability on Diets and Undernutrition of Young Children in Rural Burkina Faso,” Frontiers in Public Health 9 (2021): 693281. 34616704 10.3389/fpubh.2021.693281 PMC8489680

[35] J. Rodriguez‐Llanes , S. Ranjan‐Dash , A. Mukhopadhyay , and D. Guha‐Sapir , “Flood‐Exposure Is Associated With Higher Prevalence of Child Undernutrition in Rural Eastern India,” International Journal of Environmental Research and Public Health 13, no. 2 (2016): 210. 26861372 10.3390/ijerph13020210 PMC4772230

[36] M. Usman and K. Kopczewska , “Spatial and Machine Learning Approach to Model Childhood Stunting in Pakistan: Role of Socio‐Economic and Environmental Factors,” International Journal of Environmental Research and Public Health 19, no. 17 (2022): 10967. 36078682 10.3390/ijerph191710967 PMC9518472

[37] M. Hassan , K. Saif , M. S. Ijaz , et al., “Mean Temperature and Drought Projections in Central Africa: A Population‐Based Study of Food Insecurity, Childhood Malnutrition and Mortality, and Infectious Disease,” International Journal of Environmental Research and Public Health 20, no. 3 (2023): 2697. 36768062 10.3390/ijerph20032697 PMC9915533

[38] B. Mahapatra , T. Chaudhuri , and N. Saggurti , “Climate Change Vulnerability, and Health of Women and Children: Evidence From India Using District Level Data,” International Journal of Gynecology & Obstetrics 160, no. 2 (2023): 437–446. 36254784 10.1002/ijgo.14515

[39] H. Ishida , S. Kobayashi , S. Kanae , et al., “Global‐Scale Projection and Its Sensitivity Analysis of the Health Burden Attributable to Childhood Undernutrition Under the Latest Scenario Framework for Climate Change Research,” Environmental Research Letters 9, no. 6 (2014): 064014.

[40] A. Epstein , J. M. Torres , M. M. Glymour , D. López‐Carr , and S. D. Weiser , “Do Deviations From Historical Precipitation Trends Influence Child Nutrition? An Analysis From Uganda,” American Journal of Epidemiology 188, no. 11 (2019): 1953–1960. 31497852 10.1093/aje/kwz179 PMC7415258

[41] D. Kemajou Njatang , F. Bouba Djourdebbé , and N. D. Adda Wadou , “Climate Variability, Armed Conflicts and Child Malnutrition in Sub‐Saharan Africa: A Spatial Analysis in Ethiopia, Kenya and Nigeria,” Heliyon 9, no. 11 (2023): e21672. 38027550 10.1016/j.heliyon.2023.e21672 PMC10656247

[42] H. Randell , C. Gray , and K. Grace , “Stunted From the Start: Early Life Weather Conditions and Child Undernutrition in Ethiopia,” Social Science & Medicine 261 (2020): 113234. 32823214 10.1016/j.socscimed.2020.113234 PMC7716344

[43] K. Nicholas , L. Campbell , E. Paul , G. Skeltis , W. Wang , and C. Gray , “Climate Anomalies and Childhood Growth in Peru,” Population and Environment 43, no. 1 (2021): 39–60. 34456407 10.1007/s11111-021-00376-8 PMC8389738

[44] C. F. Dionicio López , N. Alterman , R. Calderon‐Margalit , M. Hauzer , I. Kloog , and R. Raz , “Postnatal Exposure to Ambient Temperature and Rapid Weight Gain Among Infants Delivered at Term Gestations: A Population‐Based Cohort Study,” Paediatric and Perinatal Epidemiology 36, no. 1 (2022): 26–35. 34951026 10.1111/ppe.12819

[45] J. M. Rodriguez‐Llanes , S. Ranjan‐Dash , A. Mukhopadhyay , and D. Guha‐Sapir , “Looking Upstream: Enhancers of Child Nutritional Status in Post‐Flood Rural Settings,” PeerJ 4 (2016): e1741. 26966670 10.7717/peerj.1741 PMC4782687

[46] L. S. Tusting , J. Bradley , S. Bhatt , et al., “Environmental Temperature and Growth Faltering in African Children: A Cross‐Sectional Study,” Lancet Planetary Health 4, no. 3 (2020): e116–e123. 32220673 10.1016/S2542-5196(20)30037-1 PMC7232952

[47] M. W. Cooper , M. E. Brown , S. Hochrainer‐Stigler , et al., “Mapping the Effects of Drought on Child Stunting,” Proceedings of the National Academy of Sciences 116, no. 35 (2019): 17219–17224. 10.1073/pnas.1905228116 PMC6717288 31405971

[48] B. Mahapatra , M. Walia , C. A. R. Rao , B. M. K. Raju , and N. Saggurti , “Vulnerability of Agriculture to Climate Change Increases the Risk of Child Malnutrition: Evidence From a Large‐Scale Observational Study in India,” PLoS One 16, no. 6 (2021): e0253637. 34181668 10.1371/journal.pone.0253637 PMC8238181

[49] E. I. Amondo , E. Nshakira‐Rukundo , and A. Mirzabaev , “The Effect of Extreme Weather Events on Child Nutrition and Health,” Food Security 15 (2023): 571–596.

[50] R. E. Drysdale , U. Bob , and M. Moshabela , “Coping Through a Drought: The Association Between Child Nutritional Status and Household Food Insecurity in the District of iLembe, South Africa,” Public Health Nutrition 24, no. 5 (2021): 1052–1065. 32404228 10.1017/S1368980020000105 PMC10195421

[51] E. Yeboah , N. Kuunibe , I. Mank , et al., “Every Drop Matters: Combining Population‐Based and Satellite Data to Investigate the Link Between Lifetime Rainfall Exposure and Chronic Undernutrition in Children,” Environmental Research Letters 17 (2022): 054027.

[52] B. C. Thiede and J. Strube , “Climate Variability and Child Nutrition: Findings From Sub‐Saharan Africa,” Global Environmental Change 65 (2020): 102192. 34789965 10.1016/j.gloenvcha.2020.102192 PMC8594912

[53] A. Dimitrova and J. K. Bora , “Monsoon Weather and Early Childhood Health in India,” PLoS One 15, no. 4 (2020): e0231479. 32275697 10.1371/journal.pone.0231479 PMC7147999

[54] C. N. Agabiirwe , P. Dambach , T. C. Methula , and R. K. Phalkey , “Impact of Floods on Undernutrition Among Children Under Five Years of Age in Low‐ and Middle‐Income Countries: A Systematic Review,” Environmental Health 21, no. 1 (2022): 98. 36274126 10.1186/s12940-022-00910-7 PMC9590165

[55] R. Wilts , C. Latka , and W. Britz , “Who Is Most Vulnerable to Climate Change Induced Yield Changes? A Dynamic Long Run Household Analysis in Lower Income Countries,” Climate Risk Management 33 (2021): 100330.

[56] D. Headey and M. Ruel , “Food Inflation and Child Undernutrition in Low and Middle Income Countries,” Nature Communications 14, no. 1 (2023): 5761. 10.1038/s41467-023-41543-9 PMC10505228 37717010

[57] N. Watts , M. Amann , N. Arnell , et al., “The 2018 Report of the Lancet Countdown on Health and Climate Change: Shaping the Health of Nations for Centuries to Come,” Lancet 392, no. 10163 (2018): 2479–2514. 30503045 10.1016/S0140-6736(18)32594-7 PMC7616804

[58] D. B. Rahut , R. Mishra , and S. Bera , “Geospatial and Environmental Determinants of Stunting, Wasting, and Underweight: Empirical Evidence From Rural South and Southeast Asia,” Nutrition 120 (2024): 112346. 38320385 10.1016/j.nut.2023.112346

[59] A. Sandler and L. Sun , “The Socio‐Environmental Determinants of Childhood Malnutrition: A Spatial and Hierarchical Analysis,” Nutrients 16, no. 13 (2024): 2014. 38999762 10.3390/nu16132014 PMC11243526

[60] C. Agostoni , M. Baglioni , A. La Vecchia , G. Molari , and C. Berti , “Interlinkages Between Climate Change and Food Systems: The Impact on Child Malnutrition—Narrative Review,” Nutrients 15, no. 2 (2023): 416. 36678287 10.3390/nu15020416 PMC9865989

[61] G. Danaei , K. G. Andrews , C. R. Sudfeld , et al., “Risk Factors for Childhood Stunting in 137 Developing Countries: A Comparative Risk Assessment Analysis at Global, Regional, and Country Levels,” PLoS Medicine 13, no. 11 (2016): e1002164. 27802277 10.1371/journal.pmed.1002164 PMC5089547

[62] M. Islam , S. Ali , H. Majeed , et al., “Drivers of Stunting and Wasting Across Serial Cross‐Sectional Household Surveys of Children Under 2 Years of Age in Pakistan: Potential Contribution of Ecological Factors,” American Journal of Clinical Nutrition 121, no. 3 (2025): 610–619. 39788298 10.1016/j.ajcnut.2025.01.003

[63] D. T. Desta , T. F. Teferra , and S. Gebremedhin , “The Effect of Rainfall and Temperature Patterns on Childhood Linear Growth in the Tropics: Systematic Review and Meta‐Analysis,” International Journal of Environmental Research and Public Health 21, no. 10 (2024): 1269. 39457243 10.3390/ijerph21101269 PMC11506850

[64] V. Touch , D. K. Y. Tan , B. R. Cook , et al., “Smallholder Farmers' Challenges and Opportunities: Implications for Agricultural Production, Environment and Food Security,” Journal of Environmental Management 370 (2024): 122536. 39299125 10.1016/j.jenvman.2024.122536

