# Supplementary material for: Exploring the Effects of Climate Change on Child Malnutrition: A Scoping Review
Source: J Hum Nutr Diet. 2026 Feb 26;39(2):e70220. doi: 10.1111/jhn.70220 (PMC12946572; doi:10.1111/jhn.70220)
Supplement: Supplementary file 2 — Supplementary 1 Search Strategy Klapka 2025. [file JHN-39-0-s001.pdf]

Search Strategy

Data Base	Search Query - Population terms AND Climate change terms AND Obesity terms
Pubmed (Medline)	("Population"[Title/Abstract] OR "Child*"[Title/Abstract] OR "Infant*"[Title/Abstract] OR "Preschool"[Title/Abstract]) AND ("Climate change*"[Title/Abstract] OR "Global Warming"[Title/Abstract] OR "Greenhouse gas*"[Title/Abstract] OR "Extreme Climate Event*"[Title/Abstract]) AND ("Obes*"[Title/Abstract] OR "Overweight"[Title/Abstract] OR "Body Mass Index"[Title/Abstract] OR "BMI"[Title/Abstract])
Scopus	TITLE-ABS-KEY("Population" OR "Child*" OR "Infant*" OR "Preschool") AND TITLE-ABS-KEY("Climate change*" OR "Global Warming" OR "Greenhouse gas*" OR "Extreme Climate Event*") AND TITLE-ABS-KEY("Obes*" OR "Overweight" OR "Body Mass Index" OR "BMI")
Web of Science	TS=("Population" OR "Child*" OR "Infant*" OR "Preschool") AND TS=("Climate change*" OR "Global Warming" OR "Greenhouse gas*" OR "Extreme Climate Event*") AND TS=("Obes*" OR "Overweight" OR "Body Mass Index" OR "BMI")
Embase	('Population' OR 'Child*' OR 'Infant*' OR 'Preschool')<br>AND ('Climate change*' OR 'Global Warming' OR 'Greenhouse gas*' OR 'Extreme Climate Event*')<br>AND ('Undernutrition' OR 'Underweight' OR 'Stunting' OR 'Wasting' OR 'Nutrition Deficienc*' OR 'Food Insecurit*' OR 'Food Securit*' OR 'Linear Growth')<br>AND ('Obes*' OR 'Overweight' OR 'Body Mass Index' OR 'BMI')

Data Base	Search Query - Population terms AND Climate change terms AND Undernutrition terms
Pubmed (Medline)	("Population"[Title/Abstract] OR "Child*"[Title/Abstract] OR

	"Infant*"[Title/Abstract] OR "Preschool"[Title/Abstract]) AND ("Climate change*"[Title/Abstract] OR "Global Warming"[Title/Abstract] OR "Greenhouse gas*"[Title/Abstract] OR "Extreme Climate Event*"[Title/Abstract]) AND ("Undernutrition"[Title/Abstract] OR "Underweight"[Title/Abstract] OR "Stunting"[Title/Abstract] OR "Wasting"[Title/Abstract] OR "Nutrition Deficienc*"[Title/Abstract] OR "Food Insecurit*"[Title/Abstract] OR "Food Securit*"[Title/Abstract] OR "Linear Growth"[Title/Abstract])
Scopus	TITLE-ABS-KEY("Population" OR "Child*" OR "Infant*" OR "Preschool") AND TITLE-ABS-KEY("Climate change*" OR "Global Warming" OR "Greenhouse gas*" OR "Extreme Climate Event*") AND TITLE-ABS-KEY("Undernutrition" OR "Underweight" OR "Stunting" OR "Wasting" OR "Nutrition Deficienc*" OR "Food Insecurit*" OR "Food Securit*" OR "Linear Growth")
Web of Science	TS=("Population" OR "Child*" OR "Infant*" OR "Preschool") AND TS=("Climate change*" OR "Global Warming" OR "Greenhouse gas*" OR "Extreme Climate Event*") AND TS=("Undernutrition" OR "Underweight" OR "Stunting" OR "Wasting" OR "Nutrition Deficienc*" OR "Food Insecurit*" OR "Food Securit*" OR "Linear Growth")
Embase	('Population' OR 'Child*' OR 'Infant*' OR 'Preschool') AND ('Climate change*' OR 'Global Warming' OR 'Greenhouse gas*' OR 'Extreme Climate Event*') AND ('Undernutrition' OR 'Underweight' OR 'Stunting' OR 'Wasting' OR 'Nutrition Deficienc*' OR 'Food Insecurit*' OR 'Food Securit*' OR 'Linear Growth')

**Data Base**	**Search Query - Population terms AND Climate change terms AND Undernutrition terms Obesity terms AND**
Pubmed (Medline)	("Population"[Title/Abstract] OR "Child*"[Title/Abstract] OR "Infant*"[Title/Abstract] OR "Preschool"[Title/Abstract]) AND ("Climate change*"[Title/Abstract] OR "Global Warming"[Title/Abstract] OR "Greenhouse gas*"[Title/Abstract] OR "Extreme Climate Event*"[Title/Abstract])

	AND ("Undernutrition"[Title/Abstract] OR "Underweight"[Title/Abstract] OR "Stunting"[Title/Abstract] OR "Wasting"[Title/Abstract] OR "Nutrition Deficienc*"[Title/Abstract] OR "Food Insecurit*"[Title/Abstract] OR "Food Securit*"[Title/Abstract] OR "Linear Growth"[Title/Abstract]) AND ("Obes*"[Title/Abstract] OR "Overweight"[Title/Abstract] OR "Body Mass Index"[Title/Abstract] OR "BMI"[Title/Abstract])
Scopus	TITLE-ABS-KEY("Population" OR "Child*" OR "Infant*" OR "Preschool") AND TITLE-ABS-KEY("Climate change*" OR "Global Warming" OR "Greenhouse gas*" OR "Extreme Climate Event*") AND TITLE-ABS-KEY("Undernutrition" OR "Underweight" OR "Stunting" OR "Wasting" OR "Nutrition Deficienc*" OR "Food Insecurit*" OR "Food Securit*" OR "Linear Growth") AND TITLE-ABS-KEY("Obes*" OR "Overweight" OR "Body Mass Index" OR "BMI")
Web of Science	TS=("Population" OR "Child*" OR "Infant*" OR "Preschool") AND TS=("Climate change*" OR "Global Warming" OR "Greenhouse gas*" OR "Extreme Climate Event*") AND TS=("Undernutrition" OR "Underweight" OR "Stunting" OR "Wasting" OR "Nutrition Deficienc*" OR "Food Insecurit*" OR "Food Securit*" OR "Linear Growth") AND TS=("Obes*" OR "Overweight" OR "Body Mass Index" OR "BMI")
Embase	('Population' OR 'Child*' OR 'Infant*' OR 'Preschool') AND ('Climate change*' OR 'Global Warming' OR 'Greenhouse gas*' OR 'Extreme Climate Event*') AND ('Undernutrition' OR 'Underweight' OR 'Stunting' OR 'Wasting' OR 'Nutrition Deficienc*' OR 'Food Insecurit*' OR 'Food Securit*' OR 'Linear Growth') AND ('Obes*' OR 'Overweight' OR 'Body Mass Index' OR 'BMI')
