# Supplementary material for: Exploring the Effects of Climate Change on Child Malnutrition: A Scoping Review
Source: J Hum Nutr Diet. 2026 Feb 26;39(2):e70220. doi: 10.1111/jhn.70220 (PMC12946572; doi:10.1111/jhn.70220)
Supplement: Supplementary file 3 — Supplementary Table 2. [file JHN-39-0-s002.pdf]

	First Author	Year of publication	Country of the affiliation's author	Country/Region of the study	Study Design	Study Population	Sample Size (Children)	Journal	Aim	Study period	Main Variables	Key Advantages of Variables	Presence of conflicts of interest
Seasonal droughts and the risk of childhood undernutrition in Ethiopia (15)	Anna Dimitrova	2021	USA	Ethiopia	Longitudinal	Children under five years of age	21,551	World Development	The study objectives include investigating the impact of drought exposure on childhood undernutrition in Ethiopia, understanding the effects of droughts on stunting and wasting	The study was conducted using data from three rounds of the Demographic and Health Surveys (DHS) for Ethiopia, specifically in the years 2005, 2011, and 2016	**1) Anthropometric:** height-for-age and weight-for-height<br>**2) Environmental:** Standardized Precipitation Evapotranspiration Index (SPEI)<br>**3) Socioeconomic:** Household head characteristics, Wealth, Residence type (urban/rural), Primary occupation of the household, Livelihood zone, Maternal education	**1) Anthropometric:** height-for-age and weight-for-height allows for evaluation of both chronic and acute malnutrition, providing insights into long-term growth and short-term nutritional status, helping to identify stunting and wasting.<br>**2) Environmental:** Monthly precipitation and evapotranspiration (SPEI) provide insights into climate variability and drought conditions, helping to evaluate how environmental stressors affect food availability and child health outcomes.<br>**3) Socioeconomic:** The characteristics (e.g., age, gender, and education level) of the household head often influence resource allocation, prioritization of health, and overall household welfare; Wealth reflects access to resources and services such as healthcare, education, and nutritious food, which are crucial for well-being; Urban residents generally have better access to healthcare, education, and markets, while rural residents may face infrastructural and logistical challenges; Primary Occupation of the Household: understanding occupational reliance helps assess vulnerability to external shocks, such as climate change or economic downturns, particularly in agriculture-based livelihoods; Livelihood Zone identifies how geographic and climatic conditions affect livelihood options, such as farming, fishing, or trade.	None declared
Weather shocks across seasons and child health: Evidence from a panel study in the Kyrgyz Republic (16)	Hanna Freudenreich	2022	Germany	Kyrgyzstan	Longitudinal	Children under five years of age	2010: 1,244<br>2011: 1,150<br>2012: 1,206	World Development	This study examines how cold weather shocks affect child health, particularly stunting, by exploring the role of household heating methods (electric or stove heating) and their impact on health outcomes.	The study was conducted using data from 2010 to 2012	**1) Anthropometric:** height-for-age<br>**2) Environmental:** Monthly temperature and precipitation data, Standardized Precipitation Evapotranspiration Index (SPEI), Number of excessive rainfall shocks, drought shocks and cold shocks<br>**3) Socioeconomic:** Household size, Number of children per household, Household dependency ratio, Rural/urban location, Land ownership, Agricultural income, Total household income, Main household heating source, Land size	**1) Anthropometric:** height-for-age measures of child stunting, used as indicatior of chronic malnutrition long-term child health.<br>**2) Environmental:** Rainfall and cold shocks using SPEI provide insights into the specific climatic conditions affecting food security and child health, highlighting vulnerability to extreme weather events.The SPEI is particularly useful for quantifying drought and excessive rainfall; Tracking the number of weather shocks allows the study to assess cumulative exposure<br>**3) Socioeconomic:** Household Size, Number of Children per Household, and Household Dependency Ratio: these variables indicate the distribution of resources, such as food, education, and healthcare, within a household. Larger households or high dependency ratios often face resource constraints; Land size and ownership reflect the household's agricultural productivity and food security, especially in rural areas; Agricultural Income and Total Household Income: these variables provide a snapshot of the household's financial health and reliance on specific income sources, such as agriculture; Main Household Heating Source: the type of heating source used (e.g., biomass, electricity, gas) impacts indoor air quality, carbon emissions, and health outcomes.	None declared
Heat exposure and child nutrition: Evidence from West Africa (17)	Sylvia Blom	2022	USA	Five West African countries: Benin, Burkina Faso, Côte d'Ivoire, Ghana, Togo	Cross-sectional	Children aged 3 - 36 months	32,036	Journal of Environmental Economics and Management	The aim of the study was to assess the impact of heat exposure on child nutrition, specifically focusing on stunting and wasting.	The study was conducted using data collected from 1993 to 2014	**1) Anthropometric:** height-for-age and weight-for-height<br>**2) Environmental:** Temperature exposure in hour-degree bins (heat exposure); Precipitation levels<br>**3) Socioeconomic:** Access to water source, Access to improved latrine, Household wealth quintile (proxied by asset index), Mother's education level<br>**4) Demographic:** Child's age, sex, size at birth, and firstborn status (eldest child); Mother's age and height	**1) Anthropometric:** height-for-age and weight-for-height allows for evaluation of both chronic and acute malnutrition, providing insights into long-term growth and short-term nutritional status, helping to identify stunting and wasting.<br>**2) Environmental:** Temperature Exposure in Hour-Degree Bins (Heat Exposure) provides a detailed understanding of the duration and intensity of heat exposure, which is critical for evaluating risks such as heat stress and related health outcomes; Precipitation Levels reflects the availability of water resources for agriculture, drinking, and sanitation, directly influencing food security and public health.<br>**3) Socioeconomic:** Access to water, improved latrines, and wealth quintile highlight the impact of living conditions and socioeconomic status on child nutrition and health.<br>**4) Demographic:** Factors like child's age, sex, size at birth, being the eldest child, and maternal age, education, and height provide insights into biological and socio-environmental influences on child health.	None declared
Climate change, social vulnerability and child nutrition in South Asia (18)	Kathryn McMahona	2021	USA	Bangladesh, India, Nepal, Pakistan	Cross-sectional	Children under five years of age	TotaL: 222,572<br>(Bangladesh: 21,573;<br>India: 184,177;<br>Nepal: 10,896;<br>Pakistan: 5,926)	Global Environmental Change	To examine the joint effects of temperature and precipitation anomalies during the prenatal period and first two years of life on height-for-age of children aged 2-5 in South Asia.	The study was conducted using data collected from various Demographic and Health Surveys (DHS) covering the period from 1981 to 2018	**1) Anthropometric:** height-for-age<br>**2) Environmental:** Monthly mean temperature and precipitation; Temperature and precipitation anomalies (including population-weighted anomalies and in-utero exposure)<br>**3) Socioeconomic:** Access to improved toilets; Urban-rural status; Mother's education level<br>**4) Demographic:** Mother's age at child's birth, Mother's marital status, Number of children ever born, Child's sex, Child's age, Month of birth, Place of birth, Birth order	**1) Anthropometric:** height-for-age measures of child stunting, used as indicatior of chronic malnutrition long-term child health.<br>**2) Environmental:** Monthly temperature and precipitation anomalies, particularly in in-utero exposure, impact fetal development and early childhood nutrition. Extreme climate events can disrupt food production and access, leading to malnutrition. Population-weighted anomalies helps understand how climate change affects densely populated areas, providing a broader context to child health risks.<br>**3) Socioeconomic:** Access to improved sanitation and whether a family lives in an urban or rural area greatly affect health outcomes. Poor sanitation and rural settings are linked to higher risks of infections and malnutrition. Urban areas often have better access to healthcare and nutrition, which can improve child health.<br>**4) Demographic:** Factors like the mother's age, education, and marital status shape maternal health and the child's nutritional environment. For instance, younger mothers or those with lower education levels may struggle with access to resources, affecting the child's health outcomes. The child's sex, age, and birth details provide further insights into growth patterns and<br>risks.	None declared
Child malnutrition and climate in Sub-Saharan Africa: An analysis of recent trends in Kenya (19)	Kathryn Grace	2012	USA	Kenya	Cross-sectional	Children between one and five years old	2,255	Applied Geography	The study objectives are to determine if climate variables are related to rates of childhood stunting and child hunger in Kenya, as well as to evaluate the relationship between climate variables and child malnutrition using a food security framework.	The study was conducted using data (1998 to 2008) from the Kenya Demographic and Health Survey (KDHS)	**1) Anthropometric:** height-for-age<br>**2) Environmental:** Rainfall and temperature of growing season for each year of the child's life<br>**3) Socioeconomic:** household water source, household floor material, urban or ural residence, mother's education level<br>**4) Demographic:** Child's age, sex and twin status; Mother's age and height<br>**5) Health:** birth size, breastfeeding duration, fever in past two weeks	**1) Anthropometric:** height-for-age measures of child stunting, used as indicatior of chronic malnutrition long-term child health.<br>**2) Environmental**: data on rainfall and temperature across a child's early years captures environmental impacts on food production and availability, linking climatic conditions to nutritional outcomes and vulnerability to food scarcity.<br>**3) Socioeconomic**: Water Source indicates household sanitation level, exposure to illness, and food security. Piped and well water generally suggest safer, more consistent water access, while surface water or unconventional sources may indicate higher risk of contamination and lower socioeconomic status; Floor Material serves as a proxy for wealth and living standards, where finished floors (tile, cement, wood) correlate with higher socioeconomic status and reduced risk of malnutrition; Mother's education level is linked to better health practices and nutritional knowledge;<br>**4) Demographic:** Child's age, sex, and twin status enables stratified analysis by age and sex to identify specific nutritional or health trends; Maternal age and height serves as a proxy for long-term nutritional status and can impact birth outcomes and child growth patterns.<br>**5) Health:** Birth size acts as a marker of intrauterine growth and potential early-life health risks; Breastfeeding duration reflects adherence to recommended infant feeding practices, which are crucial for growth, immunity, and overall	Conflicts of interest section not included
Environmental risk factors and child nutritional status and survival in a context of climate variability and change (20)	Kiersten Johnson	2014	USA	Four West African countries: Mali, Burkina Faso, Guinea and Benin	Longitudinal	Children under three years of age	Not applicable	Applied Geography	The aim of this study is to explore the impacts of drought and climate-driven changes in productivity on nutrition, focusing on how these changes influence household decisions and health outcomes.	The study was conducted using data from Demographic and Health Surveys (DHS): Benin (2001), Burkina Faso (2003), Guinea (2005), and Mali (2001 and 2006)	**1) Anthropometric:** height-for-age and weight-for-age<br>**2) Environmental:** Normalized Difference Vegetation Index (NDVI)<br>**3) Socioeconomic:** Level of household wealth; Mother's education; Mother's form of employment<br>**4) Health:** Hemoglobin levels (anemia); Child survival status (mortality)	**1) Anthropometric:** height-for-age and weight-for-height allows for evaluation of both chronic and acute malnutrition, providing insights into long-term growth and short-term nutritional status, helping to identify stunting and wasting.<br>**2) Environmental:** Incorporates NDVI to assess environmental factors impacting health. NDVI serves as a proxy for environmental productivity and growing conditions, which are vital for agricultural output and food security.<br>**3) Socioeconomics:** Household wealth reflects the economic capacity to access essential goods and services, such as nutritious food, healthcare, and education, directly influencing health and nutrition outcomes; Educated mothers are more likely to adopt better health practices, such as vaccination adherence, breastfeeding, and appropriate child feeding, improving overall child health outcomes; A mother's employment contributes to household income, improving the ability to access nutritious food and healthcare.<br>**4) Health:** Tracks hemoglobin levels to assess anemia which is important for understanding the nutritional deficiencies that can arise from dietary insufficiencies and infections. Also, evaluates survival status for mortality analysis Reflects whether the last born child of a rural mother who gave birth to the child within the past 3 years is currently alive.	Conflicts of interest section not included
Climate exposures and child undernutrition: Evidence from Indonesia (21)	Brian C. Thiede	2020	USA	Indonesia	Longitudinal	Children under five years of age	0 to 23 months: 4,812<br>24 to 59 months: 7,459	Social Science & Medicine	The objective of the study is to estimate the effects of temperature and precipitation variability on the linear growth and weight of Indonesian children	The study was conducted using data from the Indonesian Family Life Survey (IFLS) across five waves (1993–4, 1997–8, 2000, 2007–8, and 2014–5)	**1) Anthropometric:** height-for-age and weight-for-age<br>**2) Environmental:** Temperature, Precipitation, Monsoon onset (delay)<br>**3) Socioeconomic:** Household size, Number of household members under age five, Rural-urban status of the child's community, Farm ownership<br>**4) Demographic:** Child's sex and age, Household's age, Educational attainment (household)	**1) Anthropometric:** height-for-age and weight-for-height allows for evaluation of both chronic and acute malnutrition, providing insights into long-term growth and short-term nutritional status, helping to identify stunting and wasting.<br>**2) Environmental:** Precipitation, temperature, and monsoon onset delays on specific exposure periods (prenatal, first and second year of life) are crucial to understanding the impact of climate conditions on agriculture and, consequently, child nutrition.<br>**3) Socioeconomic:** Factors like land ownership, household size and number of household members offer an understanding of financial stability and resource availability within families, impacting food security.<br>**4) Demographic:** Data like age, sex, and education provide essential insights into the socioeconomic and family context of children, helping identify vulnerable groups.	Conflicts of interest section not included
Climate change and child malnutrition: A Nigerian perspective (22)	Eduard van der Merwe	2022	South Africa	Nigeria	Longitudinal	Children under five years of age	Not applicable	Food Policy	The aim of this study is to examine the relationship between climate change, specifically increasing temperatures and decreasing precipitation, and childhood malnutrition (stunting and underweight) in Nigeria.	The study spans three waves of data collection from the Living Standards Measurement Study - Integrated Surveys on Agriculture (2010–2011, 2012–2013, and 2015–2016)	**1) Anthropometric:** height-for-age, weight-for-height and weight-for-age<br>**2) Environmental:** Average monthly maximum near-surface temperature (ºC); Total monthly precipitation (mm); soil quality<br>**3) Socioeconomic:** Household size, Wealth status, Access to electricity, Household head's education level, Agriculture extension services, Access to credit or loans, nº meals provided to children; Distance freshwater, cities and markets	**1) Anthropometric:** height-for-age, weight-for-height, and weight-for-age provides detailed insights into children's nutritional status, such as stunting, wasting and underweight.<br>**2) Environmental**: Climate data relevant to agricultural productivity and food availability, which can indirectly affect malnutrition risk.<br>**3) Socioeconomic:** Household Size and Number of Meals Provided to Children indicates the availability of food and other resources for each household member, particularly children; Wealthier households or those with access to financial resources are better equipped to invest in health, education, and productive activities; Higher education levels correlate with improved management of household resources, health practices, and agricultural productivity; Access to electricity improves household conditions, enabling better lighting, food storage, and communication; Proximity to markets and cities facilitates trade, employment opportunities, and access to healthcare and education; Distance to freshwater sources reflects the availability and accessibility of clean water, a critical factor for health and nutrition.	None declared
Climate and location as determinants of childhood stunting, wasting, and overweight: An application of semiparametric multivariate probit model (23)	Alfred Ngwira	2020	Malawi	Malawi	Cross-sectional	Children under five years of age	5,149	Nutrition	The aim of this study was to investigate the effects of climate and location while using the multivariate model of malnutrition.	The study was conducted using data from the 2015 Malawi Demographic Health Survey	**1) Anthropometric:** height-for-age and weight-for-height<br>**2) Environmental:** Annual mean temperature, Annual rainfall, Vegetation (enhanced vegetation index) and length of growing season<br>**3) Socioeconomic:** mother's education level, mother's BMI, wealth status, district of residence, proximity to water body	**1) Antropometric:** height-for-age and weight-for-height allows for evaluation of both chronic and acute malnutrition, providing insights into long-term growth and short-term nutritional status, helping to identify stunting and wasting.<br>**2) Environmental:** annual temperature, rainfall, growing season length, and enhanced vegetation index (EVI) capture crucial climatic and proctivity conditions impacting food production and resource availability, linking environmental stressors directly to child health outcomes.<br>**3) Socioeconomic:** Mother's education level is linked to better health practices and nutritional knowledge; Maternal BMI can indicate her nutritional status and potential health risks (e.g., undernutrition, obesity) that may affect fetal development and child health; Wealth status is a strong determinant of access to healthcare, nutrition, and overall living conditions, which directly affect child health outcomes; Residential location can reveal regional disparities in healthcare availability, nutrition, sanitation, and public health infrastructure, influencing child health; Proximity to water bodies can indicate potential risks related to water quality and sanitation, which directly impact child health, including the risk of waterborne diseases and malnutrition.	Conflicts of interest section not included
Climate change, crop production and child under nutrition in Ethiopia; A longitudinal panel study (24)	Seifu Hagos	2014	Ethiopia	Ethiopia	Longitudinal	Children under five years of age	145	BMC Public Health	The aims of the study were to evaluate the spatial distribution of rainfall, temperature, per capita crop availability and undernutrition; characterize the pattern and interrelationship of rainfall, temperature and under nutrition; and analyze the variations in effects across different agro ecological zones of Ethiopia.	The study was conducted with secondary data over the period of 1996 to 2004.	**1) Anthropometric:** height-for-age, weight-for-height and weight-for-age<br>**2) Environmental:** Monthly rainfall (mm), Monthly temperature (°C), Altitude, Per capita crop production (cereals and oilseeds) (kg), Livestock ownership/production<br>**3) Demographic:** Projected population	**1) Anthropometric:** height-for-age, weight-for-height, and weight-for-age provides detailed insights into children's nutritional status, such as stunting, wasting and underweight.<br>**2) Environmental:** monthly rainfall, altitude, temperature affects agricultural output, water availability, and overall food security. Rainfall impacts crop production, altitude may result in shorter growing seasons, limited crop diversity, and lower agricultural productivity. Futhermore, temperature influences both the growing season and crop viability.<br>**3) Demographic:** Projected population data helps anticipate future population growth or decline, aiding in the planning of healthcare, education, and other essential services, ensuring resources are adequately distributed.	None declared
Characterizing the contribution of high temperatures to child undernourishment in Sub-Saharan Africa (25)	Rachel E. Baker	2020	USA	Sub-Saharan Africa (30 countries): Burundi, Cameroon, Central African Republic, Democratic Republic of Congo, Rwanda, Ethiopia, Kenya, Madagascar, Malawi, Somalia, Tanzania, Uganda, Lesotho, Namibia, Swaziland, Zambia, Zimbabwe, Benin, Burkina Faso, Cote d'Ivoire, Ghana, Guinea, Liberia, Mali, Niger, Nigeria, Senegal, Sierra Leone, Togo	Cross-sectional	Children between one and five years old	192,000	Scientific Reports	To investigate the relationship between temperature and child nutrition outcomes, estimate the effects of increased temperatures on child nutrition over different time scales, and project the impact of future warming on malnutrition in Sub-Saharan Africa.	Projections for each month from 2015 to 2100	**1) Anthropometric:** height-for-age, weight-for-height and weight-for-age<br>**2) Environmental:** Monthly temperature and precipitation data; Climate change projections from CMIP5 models<br>**3) Socioeconomic:** Rural or urban location, Country-level projections (World Bank)	**1) Anthropometric:** height-for-age, weight-for-height, and weight-for-age provides detailed insights into children's nutritional status, such as stunting, wasting and underweight.<br>**2) Environmental:** Monthly temperature and precipitation data give a fine temporal resolution of environmental conditions, enabling detailed analyses of seasonal and climatic impacts on child health. Future projections of climate change available trough CMPIP5 providies insights into potential increases inchild malnutrition due to rising temperatures.<br>**3) Socioeconomic:** Country-Level Projections from the World Bank: these projections provide robust, country-specific socioeconomic data, enabling context-aware analysis of health trends by accounting for population growth, economic conditions, and development indicators over time. Additionally, the differentiation between rural and urban areas allows for a comparative analysis of the effects on these two distinct groups.	None declared
Climate, weather and child health in Burkina Faso (26)	Shouro Dasgupta	2023	Italy	Burkina Faso	Longitudinal	Children under five years of age	Not applicable	Australian Journal of Agricultural and Resource Economics	To provide a comprehensive assessment of the impact of climate and weather shocks on child health (mortality, stunting, wasting) in Burkina Faso	The study was conducted using data from multiple waves of the Demographic and Health Surveys (DHS), covering the period from 1993 to 2010	**1) Anthropometric:** height-for-age, weight-for-height<br>**2) Environmental:** Mean temperature (°C); Precipitation variability (mm); Lifetime exposure to temperature; SPEI (Standardized Precipitation Evapotranspiration Index)<br>**3) Socioeconomic:** International Wealth Index (IWI), Household income and wealth, Household size, Educational attainment of the mother, Access to electricity, Access to water, Quality of housing and sanitation, Type of flooring material, Cooking fuel used<br>**4) Demographic:** Child's mortality	**1) Anthropometric:** height-for-age and weight-for-height allows for evaluation of both chronic and acute malnutrition, providing insights into long-term growth and short-term nutritional status, helping to identify stunting and wasting.<br>**2) Environmental:** Data on lifetime temperature exposure, monthly temperature, precipitation variability, and the Standardized Precipitation-Evapotranspiration Index (SPEI) are essential for understanding how climate variability impacts agriculture, food security, and overall health conditions, particularly in climate-sensitive regions.<br>**3) Socioeconomic:** Variables like household income, wealth, size, maternal education, and access to electricity, water sanitation, flooring quality, and cooking fuel shed light on living conditions, resource availability, and potential health risks, all of which significantly influence child well-being.<br>**4) Demographic:** Tracking child mortality rates gives insights into overall child health and survival, which reflect broader health and socioeconomic conditions affecting children.	Conflicts of interest section not included
Climate-related child undernutrition in the lake Victoria basin: an integrated spatial analysis of health surveys, ndvi, and precipitation data (27)	David López-Carr	2016	USA	Eastern Africa's Lake Victoria Basin countries: Burundi, Kenya, Rwanda, Tanzania, and Uganda	Ecological	Children under five years of age	856	IEEE Journal of Selected Topics in Applied Earth Observations and Remote Sensing	The study aims to develop a spatially and temporally integrative dataset of climate exposure components related to children's vulnerability to undernutrition and map measures of climate-related vulnerability to undernutrition among children under 5 in Eastern Africa's LVB.	1. Anthropometric data: 2008-2012<br>2. Monthly rainfall data: 1981-2012<br>3. NDVI data: 1982-2012	**1) Anthropometric:** height-for-age<br>**2) Environmental:** Monthly rainfall; Normalized Difference Vegetation Index (NDVI)	**1) Anthropometric:** height-for-age measures of child stunting, used as indicatior of chronic malnutrition long-term child health.<br>**2) Environmental:** Monthly rainfall and NDVI reflect agricultural productivity, food security, and ecosystem health. NDVI measures vegetation and crop health, while rainfall indicates water availability, both influencing food supply and child nutrition.	Conflicts of interest section not included
Projecting the impact of air pollution on child stunting in India-synergies and trade-offs between climate change mitigation, ambient air quality control, and clean cooking access (28)	Asya Dimitrova	2022	Germany	India	Observational and Modeling	Children under five years of age	203,870	Environmental Research Letters	The study investigates how climate change mitigation, air pollution control, and energy access policies impact child linear growth in India.	The study was conducted using data from 2009 to 2016	**1) Anthropometric:** height-for-age<br>**2) Environmental:** Annual average PM2.5 concentrations, In-utero ambient PM2.5 exposure, Household cooking fuel type, Type of primary cooking fuel of the households<br>**3) Socioeconomic:** Household income levels, Urban/rural residence, Caste, Maternal education	**1) Anthropometric:** height-for-age measures of child stunting, used as indicatior of chronic malnutrition long-term child health.<br>**2) Environmental:** Annual average PM2.5 concentrations measure air pollution's impact on health, particularly respiratory and growth outcomes. Type of primary cooking fuel identifies household energy sources contributing to indoor air pollution and its health risks. In-utero ambient PM2.5 exposure is important for understanding the impact of air pollution on child health; Uptake of clean cooking fuels links socio-economic status to health outcomes by reducing inner household air pollution exposure.<br>**3) Socioeconomic:** Household Income Levels reflects the household's capacity to afford essential goods and services, such as nutritious food, healthcare, and education; Urban/Rural Residence: urban areas typically have better access to healthcare, sanitation, and education, whereas rural areas may face challenges in terms of infrastructure and service availability; Caste reflects historical social hierarchies that often influence access to resources, healthcare, and education, affecting overall well-being and opportunities.	None declared

	First Author	Year of publication	Country of the affiliation's author	Country/Region of the study	Study Design	Study Population	Sample Size (Children)	Journal	Aim	Study period	Main Variables	Key Advantages of Variables	Presence of conflicts of interest
Child malnutrition and recurrent flooding in rural eastern India: a community-based survey (29)	Jose Manuel Rodriguez-Llanes	2011	Belgium	India	Cross-sectional	Children aged 6–59 month	Flooded villages: 191<br>Non-flooded villages: 161	BMJ Open	This study aims to improve the understanding of the relationship between exposure to floods and malnutrition in children aged 6-59 months in rural India.	The study was conducted between October 6 and November 19, 2008.	**1) Anthropometric:** height-for-age, weight-for-height and weight-for-age<br>**2) Environmental:** Child exposure to flood, measured whether the village were flooded or not<br>**3) Socioeconomic:** household size, number of children under 5 years in household, main occupation of the household, religion, caste, household income, source of drinking water, storage of cooked food<br>**4) Health:** Immunisation coverage	**1) Anthropometric:** height-for-age, weight-for-height, and weight-for-age provides detailed insights into children's nutritional status, such as stunting, wasting and underweight.<br>**2) Environmental:** Tracking whether a child's village experienced flooding offers a measure of vulnerability to environmental shocks, which can impact food security, health, and sanitation.<br>**3) Socioeconomic:** household size, the number of young children, main occupation, religion, income, water source, and food storage methods provide insight into living conditions, resource availability, and potential health risks, population vulnerability, all of which affect child nutrition and health.<br>**4) Health:** Immunisation coverage reflects access to and utilization of preventive healthcare services and provides a measure of how well a population is shielded from vaccine-preventable diseases that can significantly impact child growth and development.	None declared
Climate change, crop yields, and undernutrition: Development of a model to quantify the impact of climate scenarios on child undernutrition (30)	Simon J. Lloyd	2011	UK	Multinational (five regions in South Asia and sub-Saharan Africa).<br>South Asia: Bangladesh, Bhutan, India, Nepal, Pakistan.<br>Sub-Saharan Africa, East: Burundi, Djibouti, Ethiopia, Kenya, Madagascar, Malawi, Mozambique, Rwanda, Uganda, United Republic of Tanzania , Zambia<br>Sub-Saharan Africa, West: Benin, Burkina Faso, Cameroon, Chad, Cote d'Ivoire, Gambia, Ghana, Guinea, Guinea-Bissau, Liberia, Mali, Mauritania, Niger, Nigeria, Senegal<br>Sub-Saharan Africa, Central: Angola, Central African Republic, Congo, Democratic Republic of the Congo, Gabon<br>Sub-Saharan Africa, South: Botswana, Lesotho, Swaziland,	Longitudinal	Children under five years of age	Not applicable	Environmental Health Perspectives	The aims of the study were to developed a model for estimating future undernutrition that accounts for food and nonfood (socioeconomic) causes and can be linked to available regional scenario data and also to estimated child stunting attributable to climate change in five regions in South Asia and sub-Saharan Africa (SSA) in 2050	The study was conducted using data from the period 1988–2008, with projections extending to 2050.	**1) Anthropometric:** height-for-age<br>**2) Environmental:** Future climate change projection scenarios (no climate change, NCAR model, CSIRO model); Climate change effects on calorie availability (via changes calorie avaliability divided by projected population)<br>**3) Socioeconomic:** Per capita Gross Domestic Product (GDP); Gini coefficient (income inequality)<br>**4) Nutritional:** Country-level undernourishment estimate, Food access	**1) Anthropometric:** height-for-age measures of child stunting, used as indicatior of chronic malnutrition long-term child health.<br>**2) Environmental:** These projection scenarios provide different potential future climates, helping to assess how various levels of climate change could impact food security, health, and overall environmental conditions; This measure allows for a better understanding of how climate change could alter food production and its distribution relative to population growth, directly influencing calorie availability per person.<br>**3) Socioeconomic:** GDP per capita is a crucial measure of a country's overall economic performance, reflecting the average income of its citizens and their potential access to resources such as food, healthcare, and education; The Gini coefficient quantifies income distribution within a country, indicating the level of inequality.<br>**4) Nutritional:** This estimate provides a clear picture of the extent of undernourishment within a country, highlighting potential food security challenges and the need for interventions to ensure adequate nutrition; Food access refers to the availability of sufficient, nutritious, and affordable food for a population.	None declared
A global-level model of the potential impacts of climate change on child stunting via income and food price in 2030 (31)	Simon J.Lloyd	2018	UK	Multinational (44 countries): Albania, Armenia, Bangladesh, Bolivia, Bosnia & Herzegovina, Burkina Faso, Cambodia, Cameroon, China, Colombia, Cote d'Ivoire, Dominican Republic, Egypt, El Salvador, Ghana, Guatemala, Honduras, India, Indonesia, Jamaica, Kenya, Kyrgyzstan, Lao PDR, Lesotho, Madagascar, Malawi, Mauritania, Mexico, Mongolia, Mozambique, Namibia, Nepal, Nicaragua, Niger, Pakistan, Peru, Romania, Rwanda, Senegal, Sierra Leone, Sri Lanka, Swaziland, Tajikistan, Tanzania, TFYR of Macedonia, Turkey, Uzbekistan, Vietnam, Zambia.	Longitudinal	Children under five years of age	Not applicable	Environmental HealthPerspectives	The objective of the study is to adopt a new perspective to assess how climate change may affect child stunting through its impacts on two interacting socioeconomic factors: the incomes of the poorest 20% of the population (influenced by climate impacts on crop production, health, labor productivity, and disasters) and food prices.	Projections of the potential impacts of climate change on child stunting in 2030	**1) Anthropometric:** height-for-age<br>**2) Environmental:** Climate and growth scenarios (RCP2.6, RCP8.5, SSP4, and SSP5 pathways)<br>**3) Socioeconomic:** Income for the poorest 20% (Per capita, in PPP, rural and urban); Ratio of rural to urban income for the poorest 20%; Deflated Food Consumer Price Index (fCPI/gCPI)	**1) Anthropometric:** height-for-age measures of child stunting, used as indicatior of chronic malnutrition long-term child health.<br>**2) Environmental:** The use of Representative Concentration Pathways (RCPs) combined with Shared Socioeconomic Pathways (SSPs) enables evaluation of potential future climate impacts on nutrition.<br>**3) Socioeconomic:** Variables that highlight socioeconomic vulnerability and disparity between rural and urban income.	None declared
Global warming may significantly increase childhood anemia burden in sub-Saharan Africa (32)	Yixiang Zhu	2023	China	Sub-Saharan Africa (26 countries). Eastern Africa: Burundi, Ethiopia, Malawi, Mozambique, Rwanda, Tanzania, Uganda, Zambia, Zimbabwe. Central Africa: Angola, Cameroon, Congo Democratic Republic, Gabon. Western Africa: Benin, Burkina Faso, Cote d'Ivoire, Gambia, Ghana, Liberia, Nigeria, Senegal, Togo. Southern Africa: Eswatini, Lesotho, Namibia, South Africa.	Cross-sectional	Children under five years of age	275,377	One Earth	This study aims to examine the association between annual temperature and the prevalence of childhood anemia in sub-Saharan Africa	The study was conducted with data from 2003 to 2020	**1) Anthropometric:** BMI<br>**2) Environmental:** Daily mean temperature, Daily cumulative precipitation, Temperature projections under various climate change scenarios<br>**3) Socioeconomic:** Type of residence (urban/rural), Number of children in the household, Type of water infrastructure, Type of sanitation infrastructure, Floor material, Roof material, Household wealth level, Mother's education level<br>**4) Health:** Hemoglobin concentration (Hb levels, anemia status); Presence of malaria	**1) Anthropometric:** including body mass index,measures nutritional status, key for understanding child health risks.<br>**2) Environmental:** Daily Mean Temperature reflects exposure to heat stress or cold conditions, which can directly affect health outcomes, including vulnerability to heat-related illnesses and respiratory infections; Daily Cumulative Precipitation indicates water availability for agriculture, drinking, and sanitation, which directly influences food production and public health; Temperature Projections Under Various Climate Change Scenarios Highlights potential impacts of climate change on food systems, water resources, and population health, informing mitigation efforts.<br>**3) Socioeconomics:** Reflects disparities in living conditions, healthcare access, and resource availability.<br>**4) Health:** Hemoglobin concentration levels are used as proxy for anemia. Also presence of malaria (antigen detected by	None declared
Epigenetic mechanisms underlying the association between maternal climate stress and child growth: characterizing severe drought and its impact on a Kenyan community engaging in a climate change-sensitive livelihood (33)	Bilinda Straight	2022	USA	Kenya	Longitudinal	Children in utero	Drought-exposed children: 104<br>Unexposed same-sex siblings: 109	Epigenetics	This study aims to examine potential differential DNA methylation (DNAm) patterns in children exposed to severe drought in utero compared to unexposed children.	The study was conducted between 2017 and 2019	**1) Anthropometric and adiposity:** height-for-age and weight-for-age; tibia-length-for-age, triceps-skinfold-thickness-for-age, subscapular-skinfold-thickness-for-age, suprailiac-skinfold-thickness-for-age<br>**2) Environmental:** In utero exposure to drought (Historically low rainfall, High losses of livestock, Widespread food insecurity necessitating international humanitarian interventions)<br>**3) Socioeconomic:** Livestock wealth, Parental education<br>**4) Health:** cell-type proportion, Epigenome-wide DNA methylation (DNAm)	**1) Anthropometric:** Includes height-for-age, tibia-length-for-age, weight-for-age, to assess growth and nutritional status. Skinfold thicknesses (triceps, subscapular, and suprailiac) are measures adiposity, in this context a important energy body store that can support linear growth during adverse conditions.<br>**2) Environmental:** Examines in utero drought exposure, characterized by historically low rainfall, high livestock losses, and widespread food insecurity, which prompted international humanitarian interventions. This captures the environmental stresses that may affect child health outcomes.<br>**3) Socioeconomic:** Measures livestock wealth, providing a socioeconomic indicator tied to rural and agricultural livelihoods.	None declared
The Impact of Rainfall Variability on Diets and Undernutrition of Young Children in Rural Burkina Faso (34)	Isabel Mank	2021	Germany	Burkina Faso	Longitudinal	Children aged 7-60 months	1,439	Frontiers in Public Health	The study aims to investigate how rainfall variability in rural Burkina Faso affects children's diets and, in turn, their nutritional status.	The study was conducted using data from 2017 to 2019	**1) Anthropometric:** height-for-age and weight-for-height<br>**2) Environmental:** Daily rainfall data<br>**3) Socioeconomic:** Household wealth quintile, Household wealth quintile, Mother's education, Household head's education, Mother's ethnicity, Household head's ethnicity<br>**4) Nutritional:** Food intake frequency during lean and rainy seasons (past 7 days)<br>**5) Health:** History of diarrhea (past 2 weeks), History of fever (past 2 weeks), Currently breastfeeding	**1) Anthropometric:** height-for-age and weight-for-height allows for evaluation of both chronic and acute malnutrition, providing insights into long-term growth and short-term nutritional status, helping to identify stunting and wasting.<br>**2) Environmental:** Daily rainfall data (ground-base and satellite observations) helps link weather patterns to food availability and child health, providing insights into how environmental changes affect nutrition.<br>**3) Socioeconomic:** Household wealth is an essential variable to understand economic barriers to nutrition and healthcare access.<br>**4) Nutrition:** Reflects the child's food intake and dietary habits, enabling the assessment of seasonal changes in nutrition due to environmental factors like rainfall. Food intake frequency of children using a food frequency questionnaire (FFQ).	None declared
Flood-exposure is associated with higher prevalence of child Undernutrition in rural Eastern India (35)	Jose Manuel Rodriguez-Llanes	2016	Belgium	India	Cross-sectional	Children aged 6–59 month	879	International Journal of Environmental Research and Public Health	This study aims to examine the association between flooding and child undernutrition and assess children affected by the 2008 flood and by repeated flooding in 2006 and 2008	The study was conducted in September 2009, one year after significant flooding events in 2008	**1) Anthropometric:** height-for-age, weight-for-height and weight-for-age<br>**2) Environmental:** Exposure to flooding (categories: non-flooded, flooded once, flooded twice)<br>**3) Socioeconomic:** Household wealth, Land ownership, Access to healthcare and public health services, Income and credit practices, Water and sanitation, Number of individuals eating from the same kitchen, Religion and caste<br>**4) Food/Nutritional:** Household experienced food shortage due to flooding, Mean number of daily meals, Mean weekly vegetable consumption, Crop and grain stock	**1) Anthropometric:** height-for-age, weight-for-height, and weight-for-age provides detailed insights into children's nutritional status, such as stunting, wasting and underweight.<br>**2) Environmental:** Evaluates exposure to flooding, which directly disrupt food security, spread disease, and compromise infrastructure, highlighting climate change's immediate effects on vulnerable populations.<br>**3) Socioeconomic:** Examines wealth, healthcare access, income, sanitation, and food consumption patterns, linking social and economic conditions helps to understand health disparities and resilience against climate shocks.<br>**4) Food/Nutritional:** Household Experienced Food Shortage Due to Flooding and Crop and Grain Stock Washed Away: These variables highlight how environmental disasters, such as flooding, disrupt food production and storage, leading to	None declared
Spatial and Machine Learning Approach to Model Childhood Stunting in Pakistan: Role of Socio-Economic and Environmental Factors (36)	Muhammad Usman	2022	Poland	Pakistan	Cross-sectional	Children under five years of age	Not applicable	International Journal of Environmental Research and Public Health	The study examines the spatial impact of socio-economic and climate factors on childhood stunting in Pakistan.	The study was conducted using data from the 2017-18 Multiple Indicator Cluster Survey (MICS)	**1) Anthropometric:** height-for-age<br>**2) Environmental:** Climate vulnerability by district (including susceptibility to floods and droughts)<br>**3) Socioeconomic:** Mean household size, Multi-dimensional poverty index (covering education, health, and standard of living), Female literacy rate, Women's exposure to mass media, Marriage before age 15<br>**4) Health:** Pre-mature birth rate, Full immunisation coverage	**1) Anthropometric:** height-for-age measures of child stunting, used as indicatior of chronic malnutrition long-term child health.<br>**2) Environmental:** Climate vulnerability to natural hazards such as floods and droughts indicates environmental risks that could disrupt food systems, healthcare access, and living conditions, thereby impacting child health and nutrition.<br>**3) Socioeconomic:** Mean household size and multi-dimensional poverty offer insights into the living environment and resource availability, influencing child nutrition and overall health; Women's exposure to mass media and female literacy rate reflect women's empowerment, which can influence access to information and resources that affect child health.<br>**4) Health:** High rates of premature births are associated with increased risks of neonatal mortality, developmental delays,	None declared
Mean Temperature and Drought Projections in Central Africa: A Population-Based Study of Food Insecurity, Childhood Malnutrition and Mortality, and Infectious Disease (37)	Munum Hassan	2023	Pakistan	Central Africa: Angola, Cameroon, Central African Republic, Chad, Democratic Republic of the Congo, Republic of the Congo, Equatorial Guinea, Gabon, Sao Tome and Principe	Descriptive, population-based	Children under five years of age	Not applicable	International Journal of Environmental Research and Public Health	The study objectives are to research acute food insecurity (AFI), investigate childhood malnutrition and mortality, analyze infectious disease burden, and explore drought and mean temperature projections throughout the twenty-first century.	The study was conducted using data from 1990 to 2022	**1) Anthropometric:** height-for-age and weight-for-height<br>**2) Environmental:** Standardized Precipitation Evapotranspiration Index (SPEI), Projected mean air temperature, Mean air temperature, Country-specific drought outcomes (SSP)<br>**3) Socioeconomic:** Urbanization, Economic development<br>**4) Health:** Child mortality, Pneumonia mortality rates, Disease burden of pneumonia and diarrhea, Diarrhea prevalence and incidence<br>**4) Nutrition:** Undernourishment (proportion of the population with inadequate	**1) Anthropometric:** height-for-age and weight-for-height allows for evaluation of both chronic and acute malnutrition, providing insights into long-term growth and short-term nutritional status, helping to identify stunting and wasting.<br>**2) Environmental:** Country-specific drought outcomes (SSP) and projected air temperature help assess climate-related risks to food security and health. SPEI (Standardized Precipitation-Evapotranspiration Index) captures precipitation anomalies and their potential impact on agriculture and water availability.<br>**3) Socioeconomics:** Urbanization reflects changes in living conditions, infrastructure, and access to health services. Economic development provides a measure of national and regional capacity to address health and nutrition challenges. GHI measures hunger in different levels like regional, country and global scale.	None declared
Climate change vulnerability, and health of women and children: Evidence from India using district level data (38)	Bidhubhusan Mahapatra	2022	Jordan	India	Cross-sectional	Children under five years of age	Not applicable	International Journal of Gynecology & Obstetrics	The aim of the study is to understand how climate change vulnerability is associated with women and children's health (WCH) at the district level in India.	The study used district level fact sheet data from the NFHS-5, the Indian equivalent of the Demographic and Health Survey (2019-2021).	**1) Anthropometric:** height-for-age, weight-for-height and weight-for-age<br>**2) Environmental:** Climate vulnerability index at the district level; Sensitivity (environmental sensitivity to climate impacts); Adaptive capacity (household/community ability to adapt to climate changes)<br>**3) Health:** Child's immunization status; Mother's method of protection during menstruation, contraception use, menstrual health management, number of prenatal visits, postnatal care	**1) Anthropometric:** height-for-age, weight-for-height, and weight-for-age provides detailed insights into children's nutritional status, such as stunting, wasting and underweight.<br>**2) Environmental:** Analysis of sensitivity and adaptive capacity, providing insights into how the environment impacts children's health and development.<br>**3) Health:** Child full imunization is an indicator of child health, reflecting the effectiveness of health systems in providing preventive care; Mother health are indicators such as use of modern contraceptives, prenatal care visits, and hygienic menstrual practices are may reflect women's health.	None declared
Global-scale projection and its sensitivity analysis of the health burden attributable to childhood undernutrition under the latest scenario framework for climate change research (39)	Hiroyuki Ishida	2014	Japan	Global (199 countries) from nine regions: Sub-Saharan Africa, Middle East, Latin America and the Caribbean, Brazil, Commonwealth of Independent States, South Asia and Oceania, Southeast Asia, India, China	Longitudinal	Children under five years of age	Not applicable	Environmental Research Letters	The study aims to estimate the future health burden of undernutrition in DALYs, considering uncertainties in climate conditions, socioeconomic factors (population, GDP growth), climate models, social inequality (GINI coefficient), and regression model parameters.	The study covers the period from 2005 to 2050, with data collected from various sources for the years 1990 to 2008	**1) Anthropometric:** height-for-age<br>**2) Environmental:** Climate change scenarios represented by 12 Global Circulation Models (GCMs) and Representative Concentration Pathways (RCPs); Shared Socioeconomic Pathways (SSPs)<br>**3) Socioeconomic:** Per capita GDP (country-level); GINI coefficient<br>**4) Health:** Disability-Adjusted Life Years (DALYs), DALYs attributable to childhood underweight (DAtU)	**1) Anthropometric:** height-for-age measures of child stunting, used as indicatior of chronic malnutrition long-term child health.<br>**2) Environmental:** The study utilizes 12 global circulation models (GCM's) to build Climate change scenarios (SSPs). This helps to model potential impacts of socioeconomic and environmental changes, supporting policy planning and mitigation strategies.<br>**3) Socioeconomic:** Per capita GDP and GINI coefficient provide insights into economic conditions and inequality. These factors are essential to understand access to resources, healthcare, and resilience against environmental changes.<br>**4) Health:** DALYs and DAtU estimate the burden associated with childhood underweight, offering a more profound	Conflicts of interest section not included
Do Deviations From Historical Precipitation Trends Influence Child Nutrition? An Analysis From Uganda (40)	Adrienne Epstein	2019	USA	Uganda	Longitudinal	Children under five years of age	3,223	American Journal of Epidemiology	The aim of this study is to investigate the nonlinear relationships between rainfall deviations and undernutrition by incorporating both objective and subjective measures of rainfall exposure.	The study was conducted using data from the Uganda National Panel Survey between 2009 and 2012	**1) Anthropometric:** height-for-age, weight-for-height and weight-for-age<br>**2) Environmental:** Household-reported drought/irregular rainfall; Deviations from long-term precipitation trends (measured by satellite); Monthly rainfall (raster data)<br>**3) Socioeconomic:** Nº of children under 5 years in the household, Nº of male and female adults in the household, Urban location, Wealth, Flooring and roofing materials, Ownership of consumer products, Toilet facilities, Type of drinking water source	**1) Anthropometric:** height-for-age, weight-for-height, and weight-for-age provides detailed insights into children's nutritional status, such as stunting, wasting and underweight.<br>**2) Environmental:** Self-reported drought and rainfall irregularities, combined with precise measurements from satellite, ground station, and raster rainfall data, capture local climate challenges. These insights help identify climate risks to food security and child nutrition. Deviations from long-term precipitation trends measured by satellite captures variations in rainfall that may not be reported accurately by households, thus providing a more reliable assessment of environmental conditions.	None declared
Climate variability, armed conflicts and child malnutrition in sub-saharan Africa: A spatial analysis in Ethiopia, Kenya and Nigeria (41)	Donald Kemajou Njatang	2023	Canada	Ethiopia, Kenya, Nigeria	Cross-sectional	Children under five years of age	Not applicable	Heliyon	This research aims to highlight, under the hypothesis of spatial non-stationarity, the influence of climatic variations and armed conflicts on malnutrition among children under 5 in Ethiopia, Kenya and Nigeria	The study was conducted during the period from 2010 to 2020	**1) Anthropometric:** height-for-age<br>**2) Environmental:** Average monthly rainfall, Maximum monthly temperatures, Normalized Difference Vegetation Index (NDVI)<br>**3) Socioeconomic:** Household's use of unimproved water, Women with secondary education and above, Proportion of working women, presence of Inter- or intra-state armed conflict, Non-state conflict, Unilateral violence	**1) Anthropometric:** height-for-age measures of child stunting, used as indicatior of chronic malnutrition long-term child health.<br>**2) Environmental:** Monthly rainfall, maximum monthly temperatures influence agricultural productivity and food avalability challenges. NDVI (Normalized Difference Vegetation Index) is a proxy of productivity which is related to agricultural output and food security.<br>**3) Socioeconomic:** Household access to unimproved water is critical for assessing sanitation conditions and the risk of waterborne diseases, which affect overall health.<br>Armed Conflicts: Tracking inter- and intra-state conflicts, as well as non-state conflicts and unilateral violence, allows for	None declared
Stunted from the start: Early life weather conditions and child undernutrition in Ethiopia (42)	Heather Randell	2020	USA	Ethiopia	Longitudinal	Children under five years of age	23,026	Social Science & Medicine	The aim of the study is to examine the impacts of weather conditions, specifically rainfall and temperature, on stunting among children aged one to four years in Ethiopia.	The study was conducted using data from four rounds of the Ethiopia Demographic and Health Survey (DHS) from 2000, 2005, 2011, and 2016	**1) Anthropometric:** height-for-age<br>**2) Environmental:** Mean maximum daily temperature (°C), Mean monthly rainfall (cm) (averaged across the child's prenatal period and early life)<br>**3) Socioeconomic:** Region, Urban/rural residence, Potential migration status, Mother's education	**1) Anthropometric:** height-for-age measures of child stunting, used as indicatior of chronic malnutrition long-term child health.<br>**2) Environmental:** Mean maximum daily temperature and mean monthly rainfall during the child's prenatal period and early life allow the study of how climate variables influence child growth and stunting.<br>**3) Socioeconomic:** Region, urban/rural status, and potential migration status highlight socio-economic disparities that may affect child nutrition and access to resources; Educated mothers are more likely to adopt preventive healthcare practices, ensure balanced diets, and seek timely medical care for their children.	Conflicts of interest section not included
Climate anomalies and childhood growth in Peru (43)	Khristopher Nicholas	2021	USA	Peru	Cross-sectional	Children under five years of age	69,850	Population and Environment	The aim of the study is to examine how climate anomalies, particularly rainfall variations, affect childhood growth in Peru, while considering social factors and different stages of childhood development, in order to understand the potential impact of future climate change on stunting rates.	The study was conducted with data from the Demographic and Health Survey (1996 to 2012)	**1) Anthropometric:** height-for-age<br>**2) Environmental:** monthly precipitation and monthly temperature<br>**3) Socioeconomic:** household size, wealth index, rural or urban residence, mother's education level<br>**4) Demographic:** child's age, sex, mother's age and Indigenous status (or Indigeneity)	**1) Anthropometric:** height-for-age measures of child stunting, used as indicatior of chronic malnutrition long-term child health.<br>**2) Environmental**: Monthly data on temperature and precipitation help assess how climatic conditions affect food availability, health risks, and overall child development, particularly in regions highly sensitive to environmental changes.<br>**3) Socioeconomic:** Urban/rural designation, household size, and wealth capture socioeconomic disparities that influence access to resources, healthcare, and nutrition, which are key factors in child growth and development.<br>**4) Demographic:** Variables like child's age, sex, mother's age, maternal education, and indigeneity offer essential context for understanding individual and family-level factors influencing growth and vulnerability to malnutrition.	None declared
Postnatal exposure to ambient temperature and rapid weight gain among infants delivered at term gestations: a population-based cohort study (44)	Carlos Francisco Dionicio López	2022	Israel	Israel	Longitudinal	Children under five years of age	217,310	Paediatric and Perinatal Epidemiology	The study aims to examine whether exposure to high ambient temperatures during infancy is associated with rapid infant weight gain in Israel.	The study was conducted using data from 2008 to 2013	**1) Anthropometric:** weight-for-height<br>**2) Environmental:** Annual mean ambient temperature, Annual minimum ambient temperature in the first year of life<br>**3) Demographic:** Infant sex, Maternal age, Paternal and Maternal education, Parity, Population group, District, Gestational age, Calendar year and month of birth	**1) Anthropometric:** Weight for age is a crucial indicator of overall child growth and nutritional status, reflecting both acute and chronic malnutrition.<br>**2) Environmental:** Annual Mean Ambient Temperature tracks long-term temperature trends, providing insights into global and regional climate change impacts, such as increased heat exposure and shifting weather patterns; Annual Minimum Ambient Temperature in the First Year of Life: exposure to extreme cold in infancy may increase the risk of respiratory illnesses, hypothermia, and long-term health problems.<br>**2) Demographic:** Population group and district help capture regional and socio-cultural variations affecting health and nutrition outcomes. Maternal age, maternal and paternal education, and parity provide insights into parental	None declared
Looking upstream: Enhancers of child nutritional status in post-flood rural settings (45)	Jose Manuel Rodriguez-Llanes	2016	Belgium	India	Cross-sectional	Children aged 6 - 59 months	Flooded communities: 299<br>Non-flooded communities: 385	PeerJ	The study aims to assess the effect of maternal and paternal education on child stunting and wasting in flooded and non-flooded communities	The study was conducted in September 2009, with data from 2008 flood	**1) Anthropometric:** height-for-age and weight-for-height<br>**2) Environmental:** Exposure to flooding (categorized as non-flooded and flooded)<br>**3) Socioeconomic:** Principal means of livelihood, Annual household income, Parents' level of occupation, Maternal and Paternal education level, household size (number of people), Number of children under five in the household, religion<br>**4) Demographic:** Child's age, sex, birth weight, mother's age at marriage and age at first delivery, mother's and father's age at birth of the selected child	**1) Anthropometric:** height-for-age and weight-for-height allows for evaluation of both chronic and acute malnutrition, providing insights into long-term growth and short-term nutritional status, helping to identify stunting and wasting.<br>**2) Environmental:** Being exposed to floods captures the environmental risks that may directly affect child health outcomes, especially in the context of natural disasters.<br>**3) Socioeconomic:** Principal Means of Livelihood identifies the primary source of income for households, providing context about economic stability and resource access; Annual Household Income reflects a household's ability to afford essential goods and services, including nutritious food, healthcare, and education; Parents' Level of Occupation Indicates job security, income stability, and access to benefits, which influence overall household well-being; Maternal and Paternal	None declared
Environmental temperature and growth faltering in African children: a cross-sectional study (46)	Lucy S Tusting	2020	UK	Multinational (29 countries across sub-Saharan Africa): Angola, Benin, Burkina Faso, Burundi, Cameroon, Chad, Comoros, Democratic Republic of the Congo, Cote d'Ivoire, Gabon, Ghana, Guinea, Kenya, Liberia, Madagascar, Malawi, Mali, Mozambique, Namibia, Nigeria, Rwanda, Senegal, Sierra Leone, Swaziland, Tanzania, Togo, Uganda, Zambia, Zimbabwe	Cross-sectional	Children under five years of age	656,107	Lancet Planet Health	This study aims to assess whether exposure to high temperatures in sub-Saharan Africa is associated with child growth faltering	The study was conducted using data from 52 Demographic and Health Surveys (DHS) and Malaria Indicator Surveys (MIS) from 2003 to 2016	**1) Anthropometric:** height-for-age, weight-for-height and weight-for-age<br>**2) Environmental:** Land surface temperature (LST); Precipitation (total monthly, 2000–2016); Enhanced Vegetation Index (EVI) (mean monthly, 2000–2016)<br>**3) Socioeconomic:** Water source, Sanitation facility, Floor type, Education level of household head, Wealth index, Urban/rural status, Night-time lights (proxy for regional wealth and urbanicity), Accessibility to large cities<br>**4) Health:** Child Vaccination status and Diarrhea history (past 2 weeks)	**1) Anthropometric:** height-for-age, weight-for-height, and weight-for-age provides detailed insights into children's nutritional status, such as stunting, wasting and underweight.<br>**2) Environmental:** Utilizes synoptic mean monthly daytime land surface temperature (LST), enhanced vegetation index (EVI) (a measure of vegetation health and photosynthetic activity), monthly precipitation, nighttime lights (a proxy for regional wealth and urbanization), and accessibility to large cities (2015), providing a rich environmental context for analysis.<br>**3) Socioeconomics:** Includes indicators like drinking water source, sanitation facilities, urban-rural status, floor type, and household wealth, offering a robust socioeconomic profile.	None declared
Mapping the effects of drought on child stunting (47)	Matthew W. Cooper	2019	USA	Multinational (53 countries)	Cross-sectional	Children under five years of age	584,662	Proceedings of the National Academy of Sciences of the United States of America	The aim of the study is to map areas where child nutrition is vulnerable to precipitation shocks, specifically focusing on how factors like food system sensitivity to drought and local adaptive capacity influence this vulnerability.	The study was conducted using data from 1990 to 2016	**1) Anthropometric:** height-for-age<br>**2) Environmental:** Standardized Precipitation Evapotranspiration Index (SPEI), Rainfall anomalies, Nutritional diversity of local agricultural systems, Percentage of irrigated agriculture, Mean annual precipitation, Average monthly maximum temperature<br>**3) Socioeconomic:** Household wealth, Population density, Political stability	**1) Anthropometric:** height-for-age measures of child stunting, used as indicatior of chronic malnutrition long-term child health.<br>**2) Environmental:** Mean annual precipitaiton help assess the impact of climate variability on local foodsystems. SPEI provides a comprehensive measure of water availability by accounting for both precipitation and evapotranspiration, making it a reliable indicator of agricultural stress and drought conditions. The sensitivity of the food system to shocks and adaptive capacity of households facing drought are essential factors in understanding how external environmental pressures influence child nutrition outcomes, especially during times of climate stress; Irrigation: Irrigated agriculture is less dependent on local rainfall deficits, as it uses water from distant sources.	None declared
Vulnerability of agriculture to climate change increases the risk of child malnutrition: Evidence from a large-scale observational study in India (48)	Bidhubhusan Mahapatra	2021	India	India	Observational	Children under five years of age	Stunting / wasting / underweight: 243,213<br>Anaemia: 216,049	Plos One	The study examines how climate-induced agricultural vulnerability affects child nutrition in India.	The study was conducted using data from the National Family Health Survey 4 (NFHS-4) during the period of 2015-2016	**1) Anthropometric:** height-for-age, weight-for-height and weight-for-age<br>**2) Environmental:** Climate vulnerability index (at the district level)<br>**3) Socioeconomic:** Wealth index, Religion, Caste, Place of residence, Number of household members, Mother's education<br>**4) Health:** Hb count (anaemia)	**1) Anthropometric:** height-for-age, weight-for-height, and weight-for-age provides detailed insights into children's nutritional status, such as stunting, wasting and underweight.<br>**2) Environmental:** The Climate Vulnerability Index adds a critical environmental dimension, showing how climate change and weather patterns influence nutrition and health outcomes, highlighting areas most at risk.<br>**3) Socioeconomic:** The wealth index helps assess the socio-economic status of households, which is a key determinant of access to nutrition, healthcare, and overall well-being; socio-cultural background (religion, caste) and household characteristics (size, mother's education) affect child well-being.<br>**4) Health:** Hb count (anaemia): Hemoglobin (Hb) levels are a direct marker of anemia, often caused by iron deficiency,	None declared
The effect of extreme weather events on child nutrition and health (49)	Emily Injete Amondo	2023	Germany	Uganda	Longitudinal	Children aged 6 - 59 months	1,397	Food Security	The aim of the study is to investigate how temperature and precipitation extremes affect food production, food consumption, livestock, and child health in Uganda.	The study was conducted using data from 2009 to 2014.	**1) Anthropometric:** height-for-age<br>**2) Environmental:** Dry spell, Heatwave, Monthly temperature, Seasonal rainfall amount<br>**3) Socioeconomic:** Region, Urban/rural residence, Potential migration status, Market access, Farm area, Household head's education, Mother's education, Savings, Nonfarm work, Government aid, Credit access, Selling off assets, Aid from friends and relatives<br>**4) Nutritional:** Household Dietary Diversity Score (HDDS), Consumption of calories,	**1) Anthropometric:** height-for-age measures of child stunting, used as indicatior of chronic malnutrition long-term child health.<br>**2) Environmental:** Identifies how climate factors, such as temperature and rainfall, affect food production and availability, as well as child nutrition. Also, the inclusion of dry spells (-2 SD) and heatwaves allows for the assessment of their direct impacts on food production and nutrient availability.<br>**3) Socioeconomic:** Region and Urban/Rural Residence: these variables capture the influence of geographic location and settlement type on access to resources, healthcare, education, and infrastructure; Savings, Nonfarm Work, Government Aid, Credit Access, Selling Off Assets, Aid From Friends and Relatives: these variables collectively provide a snapshot of the	None declared
Coping through a drought: the association between child nutritional status and household food insecurity in the district of iLembe, South Africa (50)	Roisin E Drysdale	2020	South Africa	South Africa	Cross-sectional	Children under five years of age	Round 1: 431<br>Round 2: 387	Public Health Nutrition	The aim of this study is to assess and compare the association between household food insecurity and child nutritional status over two time-points taking into consideration the effects of a severe drought.	The study was conducted during and after a severe drought that affected the iLembe district from 2014 to 2016.	**1) Anthropometric:** height-for-age, weight-for-height and weight-for-age<br>**2) Environmental:** Drought period: during and after<br>**3) Socioeconomic:** Household socioeconomic status; Income; Household size; Dwelling type; Water/sanitation/hygiene access; Ownership of television and fridge<br>**4) Nutritional:** food insecurity, measured using a Coping Strategies Index (CSI)	**1) Anthropometric:** height-for-age, weight-for-height, and weight-for-age provides detailed insights into children's nutritional status, such as stunting, wasting and underweight.<br>**2) Environmental:** Drought period impacts the food security and child nutrition.<br>**3) Socioeconomic:** Household Socioeconomic Status, Income and Ownership of Television and Fridge: these variables provide a direct measure of a household's financial resources, influencing their ability to access health services, nutritious food, and other essentials; The type of dwelling reflects the quality of living conditions, while household size impacts how resources, such as food, income, and healthcare, are shared among family members; Access to clean water, sanitation, and hygiene is essential for preventing waterborne diseases, such as diarrhea, and for promoting good health.	None declared

	First Author	Year of publication	Country of the affiliation's author	Country/Region of the study	Study Design	Study Population	Sample Size (Children)	Journal	Aim	Study period	Main Variables	Key Advantages of Variables	Presence of conflicts of interest
Every drop matters: Combining population-based and satellite data to investigate the link between lifetime rainfall exposure and chronic undernutrition in children under five years in rural Burkina Faso (51)	Edmund Yeboah	2022	Germany	Burkina Faso	Cross-sectional	Children under five years of age	12,919	Environmental Research Letters	This study aims to address the knowledge gap by examining the association between cumulative lifetime rainfall exposure and chronic undernutrition in children under five years old in rural Burkina Faso.	The study was conducted using data from 2011 to 2017	**1) Anthropometric:** height-for-age<br>**2) Environmental:** Average Lifetime Rainfall Exposure (ALE); Household-level monthly rainfall estimates<br>**3) Socioeconomic:** Household size; Household socioeconomic status (SES); Caregiver's age; Caregiver's literacy	**1) Anthropometric:** height-for-age measures of child stunting, used as indicatior of chronic malnutrition long-term child health.<br>**2) Environmental:** Utilizes household-level monthly rainfall estimates and average lifetime rainfall exposure, enabling analysis of long-term environmental impacts on chronic undernutrition.<br>**3) Socioeconomic:** Considers household size and household SES are important determinant of access to food and healthcare, influencing overall child nutrition and health; Younger caregivers may have less experience or access to resources, while older caregivers might face physical limitations, both affecting the quality of childcare and nutrition practices; Literate caregivers are more likely to access and understand health and nutrition information, contributing to better caregiving practices.	None declared
